# Psilocybin-induced default mode network hypoconnectivity is blunted in alcohol-dependent rats

**DOI:** 10.1038/s41398-023-02690-1

**Published:** 2023-12-14

**Authors:** Jonathan R. Reinwald, Christian N. Schmitz, Ivan Skorodumov, Martin Kuchar, Wolfgang Weber-Fahr, Rainer Spanagel, Marcus W. Meinhardt

**Affiliations:** 1 Research Group Translational Imaging, Department of Neuroimaging, Central Institute of Mental Health, Medical Faculty Mannheim, University of Heidelberg, Heidelberg, Germany; 2 Department of Psychiatry and Psychotherapy, Central Institute of Mental Health, Medical Faculty Mannheim, University of Heidelberg, Heidelberg, Germany; 3 Research Group Systems Neuroscience and Mental Health, Department of Psychiatry and Psychotherapy, University Medical Center Mainz, Johannes Gutenberg University, Mainz, Germany; 4 Department of Molecular Neuroimaging, Central Institute of Mental Health, Medical Faculty Mannheim, University of Heidelberg, Heidelberg, Germany; 5 Institute of Psychopharmacology, Central Institute of Mental Health, Medical Faculty Mannheim, University of Heidelberg, Heidelberg, Germany; 6 Forensic Laboratory of Biologically Active Substances, Department of Chemistry of Natural Compounds, University of Chemistry and Technology Prague, Prague, Czech Republic; 7 Psychedelics Research Centre, National Institute of Mental Health, Klecany, Czech Republic

**Keywords:** Addiction, Neuroscience

## Abstract

Alcohol Use Disorder (AUD) adversely affects the lives of millions of people, but still lacks effective treatment options. Recent advancements in psychedelic research suggest psilocybin to be potentially efficacious for AUD. However, major knowledge gaps remain regarding (1) psilocybin’s general mode of action and (2) AUD-specific alterations of responsivity to psilocybin treatment in the brain that are crucial for treatment development. Here, we conducted a randomized, placebo-controlled crossover pharmaco-fMRI study on psilocybin effects using a translational approach with healthy rats and a rat model of alcohol relapse. Psilocybin effects were quantified with resting-state functional connectivity using data-driven whole-brain global brain connectivity, network-based statistics, graph theory, hypothesis-driven Default Mode Network (DMN)-specific connectivity, and entropy analyses. Results demonstrate that psilocybin induced an acute wide-spread decrease in different functional connectivity domains together with a distinct increase of connectivity between serotonergic core regions and cortical areas. We could further provide translational evidence for psilocybin-induced DMN hypoconnectivity reported in humans. Psilocybin showed an AUD-specific blunting of DMN hypoconnectivity, which strongly correlated to the alcohol relapse intensity and was mainly driven by medial prefrontal regions. In conclusion, our results provide translational validity for acute psilocybin-induced neural effects in the rodent brain. Furthermore, alcohol relapse severity was negatively correlated with neural responsivity to psilocybin treatment. Our data suggest that a clinical standard dose of psilocybin may not be sufficient to treat severe AUD cases; a finding that should be considered for future clinical trials.

## Introduction

The classic psychedelic substance psilocybin is a promising treatment option for various psychiatric disorders, such as depression [1, 2], anxiety related to terminal illness [3, 4], and addiction (reviewed in ref. [5]). Although neuroscientific research has enormously stepped up efforts to understand the neurobiology of psilocybin, substantial knowledge gaps persist, particularly regarding its precise therapeutic mechanisms that may contribute to the amelioration of psychiatric symptoms. In this context, it is important to note that most studies on psilocybin’s neurobiology and neuropharmacology have primarily focused on healthy individuals. However, it is crucial to consider that the therapeutic effects of psilocybin may vary depending on the specific psychiatric disorder being treated. Different disorders could lead to variations in how the brain responds to psilocybin treatment, and this crucial aspect has been relatively understudied in prior research efforts.

Several studies in the 1960s and 70s suggested the effectiveness of psychedelic substances in treating Alcohol Use Disorder (AUD) [6–9]. More recently, a pilot clinical study with only ten subjects provided preliminary evidence for psilocybin’s efficacy in the treatment of alcohol-dependent patients, with positive effects largely maintained at the 36-week follow-up [10]. Lately, in a randomized placebo-controlled clinical trial with 95 patients, Bogenschutz et al. [11] demonstrated that the treatment with psilocybin led to a significant and rapid reduction in the percentage of heavy drinking days. It is important to note that most studies in AUD typically use higher dosages of psilocybin ([11]; second treatment step) compared to other psychiatric diseases, such as major depression [1]. Even though precise clinical dose studies for AUD treatment with psychedelic substances are lacking, the use of exceptionally high dosages in AUD clinical trials suggests a potential interaction between alcohol addiction and the effects of psilocybin treatment, underlining the ongoing need for comprehensive, system-level investigations of the neurobiology of psilocybin in health and disease.

In healthy volunteers, neural effects of psilocybin have mainly been studied in resting-state fMRI experiments (for review see ref. [12]), which, however, show a broad heterogeneity of findings [13]. This can be explained by discrepancies in psychedelic compounds and dosages, different analytical approaches, and a variety of functional connectivity (FC) metrics [13, 14] that limit the comparability between studies [12]. One of the most robust findings is a psychedelic-induced acute decrease in FC within the default mode network (DMN) [14–19]. Aberrant DMN connectivity has also been shown for AUD patients [20] and in a rat model of AUD [21]. Possibly, such aberrant DMN activity might modify the neuronal response to psychedelic substances in AUD. However, investigations of the acute neural effects of psilocybin either in alcohol-dependent patients or in animal models of alcohol dependence are lacking.

With regard to the mentioned knowledge gaps of psilocybin’s mode of action, translational animal models provide a valuable tool to better understand the underlying mechanistic pathways in health and disease. The alcohol deprivation effect (ADE) model in long-term alcohol drinking Wistar rats is a translational approach to model alcohol relapse behavior in AUD [22], comprising compulsive drinking, craving, and loss of control in a relapse situation [22, 23]. In the ADE model, renewed access to alcohol solutions after a period of deprivation commonly leads to a pronounced (although temporary) increase in voluntary alcohol intake (i.e., the ADE). After repeated deprivation phases, the rats show an increased demand for the drug that resembles a relapse in patients with AUD [24, 25].

**Fig. 1:**
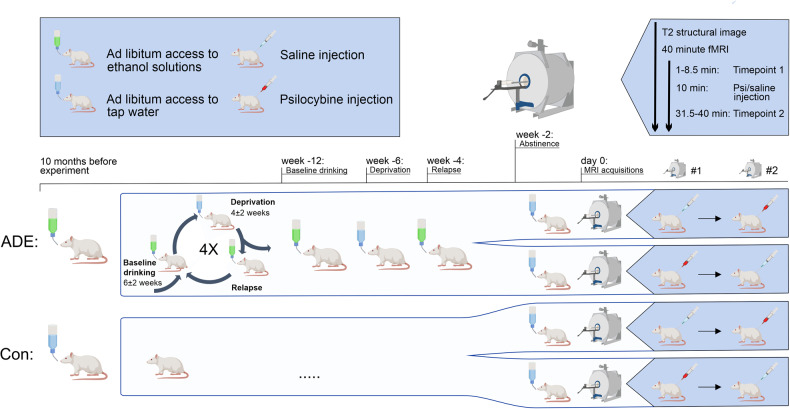
Study design. Randomized, placebo-controlled crossover study design with pharmaco-fMRI on psilocybin effects in a rat model of alcohol relapse. Two-month-old Wistar rats were used for the experiments and divided into two groups (15 ADE rats and 10 age-matched water drinking controls). The first deprivation period was introduced after twelve weeks of continuous alcohol availability. After a deprivation period (between 2–4 weeks), rats were given access to alcohol again and four more deprivation periods were introduced in a random manner. The duration of drinking and deprivation phases was irregular, i.e., approximately 6 ± 2 weeks and 4 ± 2 weeks, respectively, in order to prevent adaptive behavioral mechanisms. The long-term voluntary alcohol drinking procedure including all deprivation phases lasted for a total of 12 months. Then, all rats were assigned to two scanning sessions in a randomized order: One with psilocybin (1 mg/kg bodyweight, red syringe) and one with saline (2 ml/kg bodyweight, blue syringe). During each pharmaco-fMRI session a structural T2 weighted image was acquired followed by 36 min 30 s of functional resting-state echo-planar imaging (EPI) acquisition under an anesthesia with isoflurane and medetomidine. Each fMRI session was split into a baseline acquisition (minute 0 to 8.5), followed by the injection of psilocybin (minute 10) and the post-injection acquisition (minute 28 to minute 36.5). ADE alcohol deprivation effect rats, Con control rats, EPI echo-planar imaging, fMRI functional magnetic resonance imaging, (Created with BioRender.com).

Here, we applied a pharmaco-fMRI approach studying psilocybin in both healthy and alcohol-dependent rats to investigate general acute psilocybin-induced alterations of FC, aiming to replicate findings from human studies on a translational level. Then, we explored whether the intensity of the alcohol relapse in the ADE model is associated with the neural effects of psilocybin treatment, evaluating how a history of alcohol exposure and relapse might interfere with psychedelic-induced neural changes (Fig. 1).

## Materials and methods

### Animals, experimental design and alcohol consumption

#### Animals

Twenty-five two-month-old female Wistar rats (from our breeding colony at the Central Institute of Mental Health, Mannheim, Germany) were used for the experiments. This Wistar rat line was developed at the Max-Planck-Institute for Psychiatry in Munich (“Crl:WI(Han)” (RS:0001833)) and has been selectively bred at the Central Institute of Mental Health Mannheim for a robust alcohol drinking and ADE phenotype for over 15 years. The choice of female rats for the study was based on their lower bodyweight in comparison to males, which allows them to fit into the bore of the scanner even at the age of more than 14 months. We have previously shown [26] that female and male rats respond similarly to psilocybin treatment. Sample sizes were estimated based on a power analysis prior to study initiation in line with the 3R principle. Based on previous work [27], an effect size of d = 1.206 (subgroup by treatment interaction effect) in rs-fMRI metrics for control vs. alcohol rats with a pharmacological intervention was observed. Assuming an effect size of 1.2 for the difference between controls and ADEs (Δpost-pre), a power of 80% and α = 0.05 for a repeated measures ANOVA with within-subject (cross-over) design, we had to allocate a minimum number of 6 ADEs and 6 controls based on a calculation with the statistical power analysis program G*Power. Expecting less variance in control animals, we planned an allocation ratio of 3:2. Thus, rats were divided into two groups: 15 ADE rats and 10 age-matched water-drinking controls. All animals were housed individually in standard rat cages under a 12-h artificial light/dark cycle (lights on at 6:00 a.m.). Room temperature was kept constant (temperature: 22 ± 1 °C, humidity: 55 ± 5%). Standard laboratory rat food and water were provided ad libitum throughout the experimental period. Bodyweights were measured weekly. All experiments were approved by the institutional Committees on Animal Care and Use and by the Regierungspräsidium Karlsruhe, and were performed in accordance with the European and German national guidelines.

#### Drugs

Alcohol drinking solutions were prepared from 96% ethanol (Merck, Darmstadt, Germany) and then diluted with tap water. Psilocybin (3-[2-(dimethylamino) ethyl]−1H-indol-4-yl] dihydrogen phosphate: University of Chemistry and Technology Prague, Prague, Czech Republic) was dissolved in Ampuwa (aqua ad iniectabilia, Braun, Melsungen AG).

#### Long-term voluntary alcohol consumption with repeated deprivation phases

To model relapse-like drinking in rats, we applied the ADE model as previously described to *n* = 15 rats [26, 28]. After two weeks of habituation to the animal room, rats were given *ad libitum* access to tap water and to 5%, 10%, and 20% ethanol solutions (v/v). Spillage and evaporation were minimized by the use of special bottle caps. With this procedure the ethanol concentration remains constant for at least one week [29]. The positions of bottles were changed weekly. The first deprivation period was introduced after twelve weeks of continuous alcohol availability. After a deprivation period (between 2–4 weeks), rats were given access to alcohol again and four more deprivation periods were introduced in a random manner. The duration of following drinking and deprivation phases was irregular, i.e., approximately 6 ± 2 weeks and 4 ± 2 weeks, respectively, in order to prevent adaptive behavioral mechanisms [24]. The long-term voluntary alcohol drinking procedure including all deprivation phases lasted a total of 12 months.

To assess alcohol drinking behavior before MRI acquisition in ADE rats, baseline drinking was measured daily for the last week of a 6-week drinking period (Fig. 1). Following deprivation, alcohol bottles were reintroduced for 1 week (ADE/relapse) with daily measurements. After this final drinking phase, alcohol drinking rats were in abstinence for two weeks before scanning. Baseline alcohol consumption (g/kg bodyweight) was calculated as the mean over the last three consecutive days in the last 6-week drinking period. Relapse rates (g/kg bodyweight) were assessed on the first day of ADE in relation to baseline drinking.

#### MRI acquisition

A crossover design was used to study the effects of psilocybin on brain connectivity in ADE and control rats. Specifically, at the age of 62–68 weeks, all rats underwent two scanning sessions in a randomized order: One with psilocybin (1 mg/kg bodyweight, s.c. [30, 31]. and one with placebo (saline, 2 ml/kg bodyweight, s.c.). Four animals of the control group had to be excluded due to brain tumors detected during the structural scans, resulting in a total of *n* = 15 ADE rats and *n* = 6 control rats for further analyses.

The MRI experiments were conducted at a 9.4 T MR-scanner (Bruker BioSpec, Ettlingen, Germany) with a cryogenically-cooled rat brain coil (four-channel phased array MRI CryoProbe^TM^ and room-temperature volume transmit coil, Bruker BioSpec), providing a 2.4-fold increased signal-to-noise ratio compared to standard room temperature coils [32]. Anesthesia was performed as previously described [32], combining 0.5% isoflurane (Baxter Deutschland GmbH, Unterschleissheim, Germany) with continuous medetomidine (0.06 mg/kg/h, s.c.; Domitor, Janssen-Cilag, Neuss) after using a bolus of medetomidine (0.03 mg/kg bodyweight) and 2.5% isoflurane for initiation of the anesthesia. Importantly, this combination of light anesthetics is considered as gold standard for the assessment of resting-state networks in rats [33]. Respiratory and cardiac signals (details in Supplementary Fig. S7B–D) were recorded with 100 Hz resolution to monitor sedation depth and physiological noise for correction of rsfMRI data. The MRI acquisition protocol included: (**a**) high-resolution 3D scan (T2-weighted rapid-acquisition-with-refocused-echoes sequence, repetition time/echo time TR/TE 1200/50 ms, RARE factor 16, voxel dimensions 0.15 × 0.15 × 0.3 mm^3^); (**b**) field map (TR-short/TE-long/TE 20/1.725/5.725 ms); (**c**) rsfMRI (T2*-weighted echo-planar-imaging-free-induction-decay sequence, TR/TE 1500/17.5 ms, voxel in-plane dimension 0.365 mm, 1450 acquisitions, 30 coronal slices; slice thickness 0.5 mm). Total duration of the MRI session was 80–90 min, with a continuous rsfMRI measurement lasting 36 min and 30 s. Psilocybin (1 mg/kg bodyweight, s.c.) was administered exactly 10 min after the start of the rsfMRI acquisition. For sequences’ details see Supplement.

#### RsfMRI preprocessing

RsfMRI preprocessing and subsequent analysis steps were performed in a blinded fashion and are illustrated in Supplementary Fig. S9. Image preprocessing was performed similar to our previous studies in rodents [32, 34] and included the following steps: (**a**) correction for magnetic field inhomogeneities and movement (“realign & unwarp”, SPM12); (**b**) region-specific correction for respiratory and cardiac signals (Aztec software) [35]; (**c**) slice-timing correction to the mean slice (SPM12); (**d**) coregistration and spatial normalization to SIGMA atlas template [36] using DARTEL-based flow fields (obtained from the 3D image coregistration and normalization) (SPM12); (**e**) regression of movement parameters and cerebrospinal fluid signal (FSL, version 5, http://ww.fmrib.ox.ac.uk/fsl); (**f**) identification of motion-affected frames based on framewise displacement >0.05 mm [34] and correction with subsequent scrubbing (spline-curve interpolation) [37]; (**g**) band-pass filtering (0.01–0.1 Hz) (Analysis of Functional NeuroImages v2) [38]. No significant differences could be found for mean motion between ADE and control animals and between psilocybin and placebo, respectively (Supplementary Fig. S7A). For all animals, motion affected less than 10% of all frames and was on a very low level.

Within the crossover design, each animal underwent two rsfMRI sessions in randomized order, one with psilocybin and one with saline. For all further analyses, each of the 36 min 30 s long rsfMRI dataset was split into a first dataset prior to drug application (time point 1 (TP1), first 340 frames, 0 min–8 min 30 s) and a second dataset starting 18 min after drug application (time point 2 (TP2), last 340 frames, 28 min–36 min 30 s), with the latter capturing the effects of psilocybin or saline.

### Whole-brain functional connectivity analyses

#### Global brain connectivity

Voxel-wise global brain connectivity (GBC) is a hypothesis-free whole-brain approach which – in contrast to region-of-interest-(ROI-)based analyses – does not rely on a priori selection of a specific brain parcellation. GBC may enhance the chances of identifying pharmacologically-induced connectivity changes on a whole-brain level [14] and was recently applied in several pharmacological rsfMRI studies in humans to assess the effects of different psychedelics [14, 39]. As suggested by the literature, GBC was calculated as the average connectivity strength of each voxel with all other voxels of the brain [14, 39, 40]. First, fully-preprocessed EPI images were spatially smoothed with a 0.6 mm kernel. For the next analysis steps, a gray matter mask including all 44 cerebral atlas regions of our ROI-based approach was used, excluding cerebellum and olfactory bulb as not covered by the EPI’s field of view. For each voxel included in the mask, we computed Pearson’s correlation with every other whole-brain voxel included in the gray matter mask, transformed the correlations to Fisher z-values, and finally computed their voxel-wise mean [39]. This calculation results in GBC maps for each subject, which were fed into second-level SPM analysis to assess (1) within-subject psilocybin treatment effects and (2) subgroup-by-treatment interaction effects. GBC was calculated using custom-written in-house Matlab scripts.

#### Network-based statistic (NBS)

To assess psilocybin-induced changes in functional connectivity (FC) on a regions-of-interest (ROI)-based whole-brain level, we applied NBS, a non-parametric cluster-based method identifying potentially connected networks and controlling for multiple comparisons [41], similar to our previous studies [42, 43]. ROI-based analytical approaches are complementary to GBC, providing additional insights into brain network function. In contrast to GBC, they characterize a network based on specific connections between predefined brain regions, while GBC is more unspecific and focuses on voxel-wise average connectivity to all other voxels of the brain. First, FC matrices were calculated for TP1 and TP2 of each session separately. Specifically, mean time-courses of 44 bihemispheric brain regions from SIGMA atlas [36] were extracted and Pearson’s correlation coefficients between the time-courses were computed, resulting in individual networks, with nodes representing brain regions and edges correlation coefficients. Next, the two FC matrices were subtracted (FCmat_TP2_ − FCmat_TP1_), resulting in a difference matrix (FCmat_Diff_) for each of the two sessions (saline and psilocybin), representing the change in FC after drug or placebo application compared to the baseline before. Then, we assessed (1) within-subject treatment effects comparing the difference matrices of psilocybin to saline and (2) subgroup-by-treatment interaction effects to investigate differences of psilocybin between ADE and control animals. The statistical models were specified in terms of a general linear model with F-tests performed for each edge. A stringent primary threshold (*p*_pt_ < 0.01, *F*_1,19_ = 7.31) was used to discard sub-threshold edges. The contiguous surviving edges were defined as a cluster. The cluster extent was compared with the maximum extent resulting from 5.000 random permutations (*p*_NBS_ < 0.05). Exchange blocks were integrated into the NBS analysis to account for the paired crossover design of our data with saline and psilocybin sessions for each animal.

#### Graph analysis

**Fig. 2:**
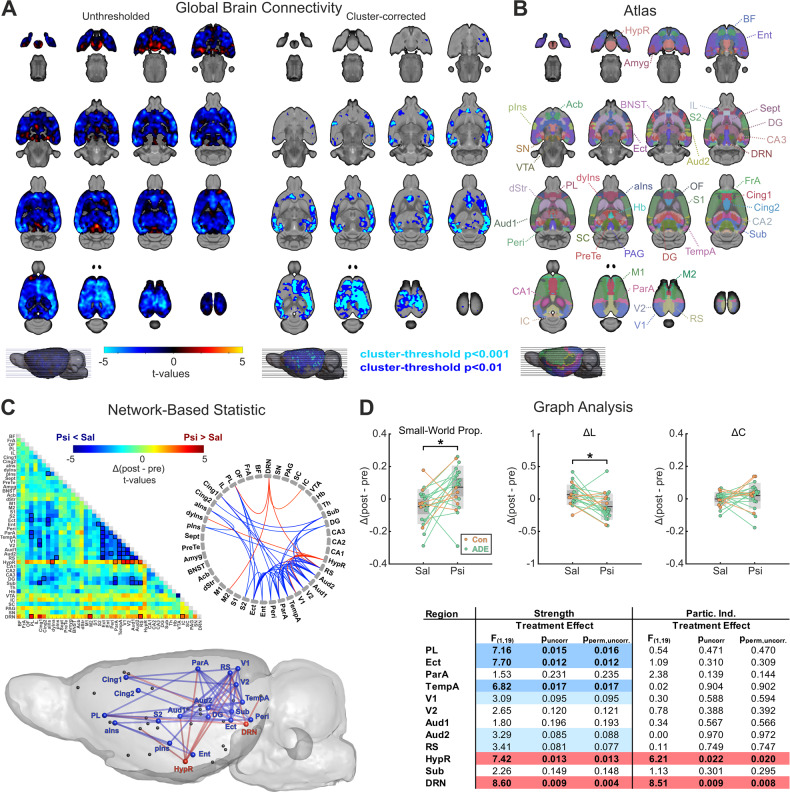
Global brain connectivity (GBC), network-based statistic (NBS) and graph analysis (GA) demonstrate psilocybin-induced treatment effects on whole-brain network functioning. **A** The left panel demonstrates an unthresholded t-value map for the post-hoc analysis of the within-subject treatment condition (starting 18 min after subcutaneous psilocybin administration) comparing psilocybin to placebo (*n* = 21 rats, crossover design). Regions exhibiting reduced GBC under psilocybin are indicated in blue to light blue, while red to yellow indicate regions with increased GBC. The right panel illustrates areas surviving cluster-correction (*p*_FWE cluster-corrected_ < 0.05) with a cluster-defining threshold of *t* > 2.53 (dark blue, cluster-size ≥ 7998 voxels, corresponding to *p*_CDT_ < 0.01) and *t* > 3.55 (light blue, cluster-size ≥ 45 voxels, corresponding to *p*_CDT_ < 0.001), respectively. For exact cluster sizes, see Supplementary Excel File “Overview_Statistics.xlsx”. **B** shows atlas regions considered for region-of-interest-based parcellation in functional MRI analyses. **C** NBS results comparing functional connectivity alterations 18 min after psilocybin administration to placebo demonstrate a pattern of strong psilocybin-induced cortical hypoconnectivity (blue), while hypothalamus and dorsal raphe nucleus revealed opposing hyperconnectivity (red, *p*_NBS_ < 0.01; primary threshold *F*_pt_ > 7.31, corresponding to *p*_pt_ < 0.01). Data are illustrated in clockwise manner in a connectivity matrix as t-values (psilocybin vs saline) with black boxes marking the connections of the cluster surviving the NBS, a connectogram and a sagittal brain view. **D** Graph analysis showed increased small-world propensity after psilocybin administration when compared to saline (*F*_1,19_ = 6.049, *p*_FDR-corrected_ = 0.024, *p*_perm,FDR-corrected_ = 0.016). The deviation of the network’s characteristic path length (ΔL, from both lattice and random networks constructed with the same number of nodes and the same degree distribution, *F*_1,19_ = 6.524, *p*_FDR-corrected_ = 0.020, *p*_perm,FDR-corrected_ = 0.014) was significantly decreased after psilocybin administration, predominantly driving the changes in small-world propensity, while no differences could be found for the deviation of the clustering coefficient (ΔC, *F*_1,19_ = 1.388, *p* = 0.254, *p*_perm_ = 0.269). The table of *F*- and *p*-values (parametric and non-parametric) for within-subject treatment effects of local graph metrics (strength and participation index, all *p*-values are uncorrected for multiple comparisons, bottom-right) derived from a repeated measures ANOVA shows a pattern of significant decrease (*p*_uncorrected_ < 0.05) upon psilocybin administration for strength in PL, Ect, and TempA, while HypR and DRN demonstrate increased strength and participation index, similar to the pattern found in NBS. Only local graph metrics with at least four significantly altered connections in the NBS are shown. Blue color indicates lower and red color indicates higher values after psilocybin administration in comparison to placebo. For a table of all local graph metrics, see Supplementary Excel File “Overview_Statistics.xlsx”, sheet “Local Graph Metrics”. BF basal fore brain, FrA frontal association cortex, OF orbitofrontal region, PL prelimbic cortex, IL infralimbic cortex, Cing1 cingulate cortex area 1, Cing2 cingulate cortex area 2, aINs agranular insular cortex, dyIns dysgranular insular cortex, pIns posterior insular cortex, Sept septum, PreTe pretectal region, Amyg amygdala, BNST bed nucleus of stria terminalis, Acb accumbens, dStr dorsal striatum, M1 primary motor cortex, M2 secondary motor cortex, S1 primary somatosensory cortex, S2 secondary somatosensory cortex, Ect ectorhinal cortex, Ent entorhinal cortex, Peri perirhinal cortex, ParA parietal associative cortex, TempA temporal associative cortex, V1 primary visual cortex, V2 secondary visual cortex, Aud1 primary auditory cortex, Aud2 secondary auditory cortex, RS retrosplenial cortex, HypR hypothalamus region, CA1-CA3 fields CA1-CA3 (cornu ammonis) of hippocampus, DG dentate gyrus, Sub subiculum, Th thalamus, Hb habenula, VTA ventral tegmental area, IC inferior colliculus, SC superior colliculus, PAG periaqueductal gray, SN substantia nigra, DRN dorsal raphe nucleus, Psi psilocybin, Sal saline, Δ(post-pre) difference between post-injection and pre-injection values, ADE alcohol deprivation effect rats (*n* = 15), Con control rats (*n* = 6), *p < 0.05.

Graph analysis was applied to assess distinct brain network features on a whole-brain level complementarily to GBC and NBS. Modeling the brain as a large complex network, graph analysis quantifies whole-brain information exchange with global metrics and characterizes the influence of individual brain regions in network communication with local metrics. Graph analysis was performed according to previous studies [32], using 44 bihemispheric regions of interest from SIGMA atlas [36], Fig. 2B). Pearson’s correlation matrices calculated from mean regional time-courses (normalized by maximum weights) were used to compute network metrics (Brain Connectivity Toolbox, version 2016-01-16) [44]. Weighted networks were created by retaining 10–50% of the strongest edges (1% step), and normalized, using random graphs preserving number of nodes, degree distribution, and connectedness [45]. First, each graph metric was calculated for every threshold; afterwards, mean metric values across thresholds were used to identify systematic effects that are not dependent on a specific threshold. To explore changes in global topology, we calculated small-world propensity (SWP), characterizing the optimal small-world organization present in healthy brain networks, and the deviation of the network’s clustering coefficient (ΔC) and characteristic path length (ΔL) from both lattice and random networks constructed with the same number of nodes and the same degree distribution [46]. While ΔC characterizes local information exchange, i.e., segregation, ΔL describes long-distance information exchange, i.e., integration. Local graph metrics such as strength, local clustering coefficient and participation index were assessed to explore regional network characteristics (see definitions in the Supplement). Psilocybin-induced changes were assessed as the difference between TP2 and TP1 and compared to placebo controls in a repeated measures analysis of variance (ANOVA, see *Statistics*).

### Hypothesis-based analysis in the default mode network (DMN)

#### Functional connectivity

Converging evidence suggests an acute decrease of connectivity within the DMN as a key feature underlying psilocybin’s mode of action [14–19]. Therefore, we specifically evaluated FC within the rats’ DMN as defined by Lu et al. [47], consisting of 12 different regions including OF, PL, IL, Cing1, Cing2, RS, TempA, ParA, Aud1, Aud2, V1, and CA1. More precisely, whole-brain connectivity matrices were reduced to the 12 DMN regions. Next, we calculated the mean connectivity within the DMN and the local within-DMN connectivity strength for all DMN nodes, which is defined as the mean connectivity of each of the 12 regions to the rest of the DMN regions, separately. Again, as in our previous analyses, psilocybin-induced changes were assessed as the difference between TP2 and TP1 and compared to placebo controls in a repeated measures ANOVA (see *Statistics*).

#### Entropy

The entropic brain hypothesis proposes that entropy of spontaneous brain activity indicates qualities of the subjective experience within any given state of consciousness and implies that treatment with psychedelic substances induce an increase of both brain entropy as well as of the subjective experience [48]. Corresponding with this, brain entropy within the DMN was calculated as a potential factor underlying psilocybin-induced network changes.

Specifically, sample entropy was used to quantify regional rsfMRI signal complexity [49–51]. After preprocessing and smoothing with a 0.6 mm voxel kernel, voxel-wise sample entropy was calculated based on the mean-subtracted voxel time-courses. In accordance with the literature [52], we chose a distance tolerance threshold of 0.2 with 3 embedding dimensions, and applied standardized Euclidean distance measures. Next, mean entropy of the entire DMN and of all 12 DMN regions was estimated by averaging the resulting whole-brain entropy maps for the specific ROIs. Sample entropy was calculated for the first time series prior to drug application (TP1, first 340 frames) and the second time series ~18 min after drug application (TP2, last 340 frames), with the latter capturing the effects of psilocybin (or placebo). Psilocybin-induced changes were assessed as the difference between TP2 and TP1 and compared to placebo controls in a repeated measures ANOVA (see *Statistics*).

#### Statistics

Repeated measures ANOVA was used to investigate (1) within-subject treatment effects (psilocybin vs placebo) and (2) subgroup-by-treatment interaction effects of global and local graph metrics, mean DMN connectivity, local within-DMN strength, and sample entropy. The repeated measures ANOVA model was determined in MATLAB using fitrm and ranova functions. Outcome measures for graph metrics, DMN connectivity and entropy were calculated as the difference between TP2 (starting 18 min after drug application, 8.5 min duration) and TP1 (baseline before psilocybin or placebo administration, 8.5 min duration), respectively, and were then defined as the within-subject responses under the two different treatment conditions (psilocybin and saline) for each animal, while the subgroup was specified as the predictor variable (independent variable, ADE or control group).

To control basic assumptions of the repeated measures ANOVA, Shapiro–Wilk [53] and Bartlett’s test [54] were applied to investigate normal distribution and equality of variance for the reported metrics (global and local graph metrics, mean DMN functional connectivity, within-DMN strength and entropy). As some of the data was not normally distributed, we additionally integrated non-parametric permutation tests and reported the results together with the traditional ANOVA results in the manuscript. Specifically, we transferred our data to R (R Core Team 2022; https://www.R-project.org/) and used the *permuco* package (https://cran.r-project.org/web/packages/permuco/index.html.[55], applying *aovperm* on the respective metrics (e.g., DMN connectivity, graph metrics, etc.). The function provides *p*-values based on permutation tests specifically developed for unbalanced mixed (between-within) ANOVA designs as in our study [56]. We performed *n* = 10.000 permutations for every metric. For a detailed summary of the statistics including the results from Shapiro–Wilk and Bartlett’s tests see Supplementary Excel File “Overview_Statistics.xlsx”. We additionally applied false-discovery rate (FDR) correction for the number of tests in the respective metrics, e.g., corrected the local graph metrics for *n* = 44 tests (44 regions investigated) and the within-DMN strength for *n* = 12 tests (12 regions investigated). *P*-values are marked as “uncorrected” or “FDR-corrected” depending on their level of significance in the manuscript. The statistical methods used for GBC and NBS are delineated in the respective paragraphs, including their correction for multiple comparisons. Testing for sequence effects in GBC, NBS, graph analysis and DMN connectivity did not reveal any significant results (*p* > 0.05), so that we did not include the sequence as a covariate in our analyses.

As the repeated measures ANOVA design only allows to assess the effects of ADE in a categorical approach, distinguishing dichotomously between control and ADE animals, we additionally integrated a dimensional analysis by correlating our fMRI metrics to the intensity of the ADE phenotype. For this analysis, we focused on the most prominent and best described FC phenotypes of psilocybin treatment, i.e., the psilocybin-induced decrease of DMN connectivity and the decrease of GBC. The ADE phenotype was quantified as the percentage alcohol consumption during relapse in comparison to baseline (normalized relapse rate). Then, Spearman’s rank correlations between normalized relapse rates and the decrease of mean DMN connectivity and within-DMN strength were calculated to assess for monotonic, but not necessarily linear associations to the relapse rate. For this analysis, the difference (ΔSal − ΔPsi) of the respective metric between saline and psilocybin treatment was used (metric_ΔSal-ΔPsi_ = (metric_TP2,Sal_ − metric_TP1,Sal_)−(metric_TP2,Psi_ − metric_TP1,Psi_)) to quantify the psilocybin-induced decrease of FC. A comparable analysis was performed for GBC, correlating voxel-wise GBC scores of the ADE rats to their normalized relapse rates. This procedure resulted in a map of correlation coefficients (ρ) that were transformed to t-values ($t=\frac{\left|{\rm{\rho }}\right|}{\sqrt{\frac{1-{{\rm{\rho }}}^{2}}{N-2}}}$) and then cluster-corrected (fsl_cluster. [57],) with a cluster-defining threshold of *t* > 2.65 (corresponding to *p* < 0.01) and *t* > 3.85 (corresponding to *p* < 0.001), respectively.

For detailed results from all our statistical analyses, see Supplementary Excel File “Overview_Statistics.xlsx”.

## Results

### Global brain connectivity (GBC), network-based statistic (NBS) and graph analysis demonstrate wide-spread psilocybin-induced connectivity alterations on a whole-brain level

Recent studies demonstrated psilocybin’s brain-wide influence on resting-state functional connectivity in humans [12, 39]. We tried to replicate these findings in rats by analogously applying GBC, a measure of voxel-wise average brain-wide connectivity strength previously used in human studies [14, 39]. GBC analysis revealed a highly significant wide-spread cluster of psilocybin-induced decreased average connectivity strength 18 min after drug administration in comparison to placebo (Fig. 2A, *p*_FWE cluster-corrected_ < 0.05; cluster-defining threshold of *t* > 2.53 (corresponding to *p*_CDT_ < 0.01, dark blue) and *t* > 3.55 (corresponding to *p*_CDT_ < 0.001, light blue), respectively). This cluster encompassed mostly cortical regions such as the insula (agranular, aIns; dysgranular, dyIns), pre- (PL), infralimbic (IL) and cingulate cortices (Cing1, Cing2), somatosensory and motor regions (S1, S2, M1), auditory and visual areas (Aud1, Aud2, V1, V2), temporal and parietal association cortices (TempA, ParA) and parts of the hippocampus (cornu ammonis, CA2; subiculum, Sub). Of note, although a psilocybin-induced increase of GBC could be detected in basal, striatal and posterior brain areas including hypothalamus (HypR), dorsal raphe nucleus (DRN) and periaqueductal gray (PAG), none of these regions survived cluster correction (Fig. 2A, *p*_FWE cluster-corrected_ > 0.05; cluster-defining threshold of *t* > 2.53 corresponding to *p*_CDT_ < 0.01 and *t* > 3.55 corresponding to *p*_CDT_ < 0.001).

Next, we assessed psilocybin’s effects with regions-of-interest (ROI)-based functional connectivity analyses, namely NBS and graph analysis. In comparison to GBC, which quantifies voxel-wise average connectivity strength to *all other voxels* of the brain, ROI-based approaches provide complementary insights into brain network function characterizing connections between predefined brain regions (e.g., DRN to PL). All ROIs are illustrated in Fig. 2B. NBS, controlling the family-wise error rate when mass-univariate testing is performed at every connection comprising the graph [41], revealed a highly significant network of psilocybin-induced resting-state FC changes in comparison to saline. Similar to GBC, this network incorporated connections between multiple cortical brain areas, but additionally included HypR and DRN (*p*_NBS_ < 0.01; primary threshold (PT) *F*_pt_ > 7.31, corresponding to *p*_pt_ < 0.01, Fig. 2C). More specifically, NBS differentiated a wide-spread subnetwork of significantly *decreased* connectivity in the cortex, reaching from prefrontal regions to posterior-parietal areas (blue connections in Fig. 2C), from a distinct focal circuit of psilocybin-induced *increased* connectivity linking the two hub regions HypR and DRN to predominantly cortical areas (red connections in Fig. 2C). Cortical regions within the hypoconnected network were comparable to the findings from the GBC, including PL, Cing1, Cing2 and insula, as well as posterior-parietal regions, such as TempA, ParA, ento- (Ent), ecto- (Ect), perirhinal (Perih), V1, V2, Aud1, Aud2, and retrosplenium (RS). In contrast, the well-localized hyperconnected circuit specifically linked the HypR to dysgranula insula (dyIns), ParA, V1, V2, and RS and the serotonergic key region DRN to PL, inferior colliculus (IC), secondary motor cortex (M2) and RS.

To further characterize psilocybin-induced alterations in brain network organization on a whole-brain level, we performed graph analysis. Upon the administration of psilocybin, we found an increase in small-world propensity (SWP) characterizing the optimal small-world organization present in healthy brains (*F*_1,19_ = 6.049, *p*_FDR-corrected_ = 0.024, *p*_perm,FDR-corrected_ = 0.016, Fig. 2D). SWP reflects the deviation of a network’s clustering coefficient (ΔC, local information exchange, segregation) and characteristic path length (ΔL, long-distance information exchange, integration) from both lattice and random networks constructed with the same number of nodes and the same degree distribution [46]. In our data, psilocybin-induced facilitation of long-distance information exchange, quantified with ΔL and reflecting a higher whole-brain integration (*F*_1,19_ = 6.049, *p*_FDR-corrected_ = 0.024, *p*_perm,FDR-corrected_ = 0.014, Fig. 2D), predominantly drove the change in SWP.

The local graph metrics strength (sum of connections for a given node) and participation index (ratio of intermodule strength to the total nodal strength), illustrated for regions with at least four significant connections within the NBS network in the table in Fig. 2D, supported and expanded our previous findings. Firstly, psilocybin’s effects on strength revealed a comparable pattern to NBS and GBC results, with significantly decreased strength in PL (blue, *F*_1,19_ = 7.164, *p*_uncorrected_ = 0.015, *p*_perm,uncorrected_ = 0.016), Ect (blue, *F*_1,19_ = 7.697, *p*_uncorrected_ = 0.012, *p*_perm,uncorrected_ = 0.012), and TempA (blue, *F*_1,19_ = 6.822, *p*_uncorrected_ = 0.017, *p*_perm,uncorrected_ = 0.017), while strength was significantly increased in HypR (red, *F*_1,19_ = 7.418, *p*_uncorrected_ = 0.013, *p*_perm,uncorrected_ = 0.013) and DRN (red, *F*_1,19_ = 8.599, *p*_uncorrected_ = 0.009, *p*_perm,uncorrected_ = 0.004). Secondly, local participation index, quantifying regional hub function by the linkage between different brain modules, was increased in HypR (red, *F*_1,19_ = 6.207, *p*_uncorrected_ = 0.022, *p*_perm,uncorrected_ = 0.020) and DRN (red, *F*_1,19_ = 8.512, *p*_uncorrected_ = 0.009, *p*_perm,uncorrected_ = 0.008), underlining their switch of function upon the administration of psilocybin. A general trend towards an increase of participation index in various regions under psilocybin (Supplementary Excel File “Overview_Statistics.xlsx”) reflected the increase of whole-brain long-distance information exchange, quantified with ΔL on a global scale.

In summary, our different analytical approaches (GBC, NBS, and graph analysis) for resting-state FC data provide converging evidence for wide-spread psilocybin-induced connectivity alterations with a large cortical cluster with reduced connectivity and a well-localized hyperconnected circuit that includes the serotonergic key region DRN and the hypothalamus.

### Psilocybin induces alterations in functional connectivity and entropy of the default-mode network (DMN)

**Fig. 3:**
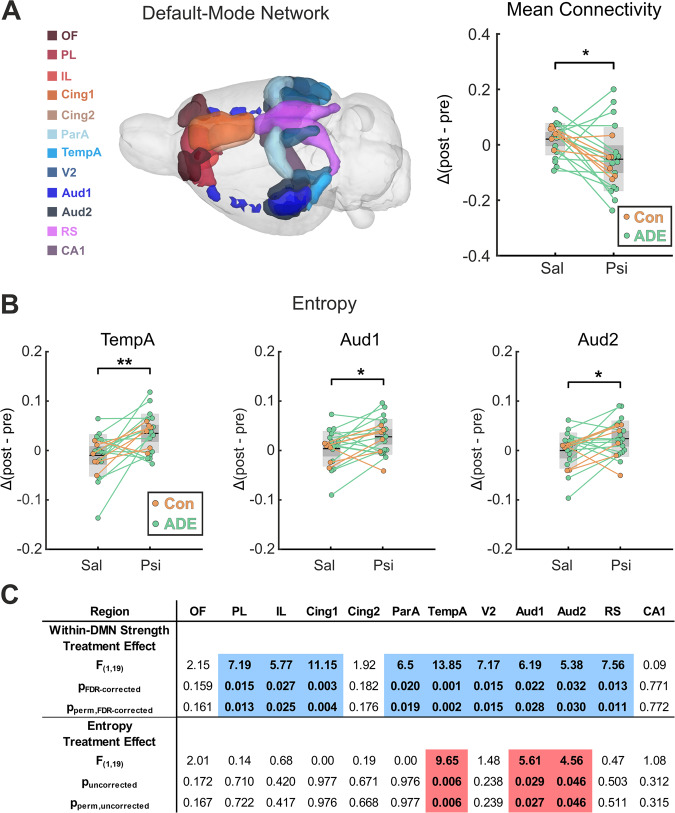
Psilocybin-induced alterations in default mode network (DMN) functional connectivity and entropy. **A** DMN was defined according to Lu et al. [47], consisting of 12 different regions including OF, PL, IL, Cing1, Cing2, RS, TempA, ParA, Aud1, Aud2, V1, and CA1, and is illustrated in the glass brain on the left. Mean functional connectivity within the DMN (top right panel) was significantly decreased after psilocybin administration when compared to placebo (*F*_1,19_ = 7.128, *p* = 0.015, *p*_perm_ = 0.016). **B** Sample entropy of the DMN regions TempA, Aud1 and Aud2 was significantly increased after psilocybin administration when compared to placebo (TempA, *F*_1,19_ = 9.654, *p*_uncorrected_ = 0.006; Aud1, *F*_1,19_ = 5.612, *p*_uncorrected_ = 0.029; Aud2, *F*_1,19_ = 4.561, *p*_uncorrected_ = 0.046; all *p*-values are uncorrected for multiple comparisons). **C** *F*-values (within-subject treatment effect) derived from a repeated measures ANOVA show a pattern of significantly decreased within-DMN strength in 9 out of 12 DMN-regions after psilocybin administration (all within-DMN strength values are FDR corrected for multiple comparisons, PL: *F*_1,19_ = 7.194, *p*_FDR-corrected_ = 0.015, *p*_perm,FDR-corrected_ = 0.013, IL: *F*_1,19_ = 5.773, *p*_FDR-corrected_ = 0.027, *p*_perm,FDR-corrected_ = 0.025, Cing1: *F*_1,19_ = 11.146, *p*_FDR-corrected_ = 0.003, *p*_perm,FDR-corrected_ = 0.004, ParA: *F*_1,19_ = 6.502, *p*_FDR-corrected_ = 0.020, *p*_perm,FDR-corrected_ = 0.019, TempA: *F*_1,19_ = 13.851, *p*_FDR-corrected_ = 0.001, *p*_perm,FDR-corrected_ = 0.002, V2: *F*_1,19_ = 7.165, *p*_FDR-corrected_ = 0.015, *p*_perm,FDR-corrected_ = 0.015, Aud1: *F*_1,19_ = 6.189, *p*_FDR-corrected_ = 0.022, *p*_perm,FDR-corrected_ = 0.028, Aud2: *F*_1,19_ = 5.377, *p*_FDR-corrected_ = 0.032, *p*_perm,FDR-corrected_ = 0.030, and RS: *F*_1,19_ = 7.575, *p*_FDR-corrected_ = 0.013, *p*_perm,FDR-corrected_ = 0.011). Blue color indicates lower and red color indicates higher values after psilocybin administration in comparison to placebo. *F*-values for the treatment effect on entropy from a repeated measures ANOVA are additionally illustrated in the lower row of the table (uncorrected for multiple comparisons). Psi psilocybin, Sal saline, Δ(post-pre) difference between post-injection and pre-injection values, ADE alcohol deprivation effect rats (*n* = 15), ANOVA analysis of variance, Con control rats (*n* = 6); ^#^*p* < 0.10; *p < 0.05; **p < 0.01. For abbreviation of brain regions, see Fig. 2. All psilocybin and saline values were first compared to its respective baseline, before feeding the difference into the repeated measures ANOVA and calculating the parametric and non-parametric *p*-values. For detailed results, see also Supplementary Excel File “Overview_Statistics.xlsx”, sheets “DMN” and “Entropy”.

One solid functional neuroimaging finding in humans is that acute psilocybin administration specifically modulates DMN connectivity [14, 16–19]. To translate this key finding to the rat brain, we further investigated functional connectivity and entropy within the DMN. DMN definition was based on previous work [47], illustrated in (Fig. 3A), dissecting DMN regions in rats under comparable resting-state conditions.

Indeed, repeated measures ANOVA results could demonstrate a significant decrease in mean DMN connectivity 18 min after psilocybin administration in comparison to placebo (*F*_1,19_ = 7.128, *p* = 0.015, *p*_perm_ = 0.016, Fig. 3A). This decline in DMN connectivity was driven by systematically decreased within-DMN strength of nine DMN regions, namely PL (*F*_1,19_ = 7.194, *p*_FDR-corrected_ = 0.015, *p*_perm,FDR-corrected_ = 0.013), IL (*F*_1,19_ = 5.773, *p*_FDR-corrected_ = 0.027, *p*_perm,FDR-corrected_ = 0.025), Cing1 (*F*_1,19_ = 11.146, *p*_FDR-corrected_ = 0.003, *p*_perm,FDR-corrected_ = 0.004), ParA (*F*_1,19_ = 6.502, *p*_FDR-corrected_ = 0.020, *p*_perm,FDR-corrected_ = 0.019), TempA (*F*_1,19_ = 13.851, *p*_FDR-corrected_ = 0.001, *p*_perm,FDR-corrected_ = 0.002), V2 (*F*_1,19_ = 7.165, p_FDR-corrected_ = 0.015, p_perm,FDR-corrected_ = 0.015), Aud1 (*F*_1,19_ = 6.189, *p*_FDR-corrected_ = 0.022, *p*_perm,FDR-corrected_ = 0.028), Aud2 (*F*_1,19_ = 5.377, *p*_FDR-corrected_ = 0.032, *p*_perm,FDR-corrected_ = 0.030), and RS (*F*_1,19_ = 7.575, *p*_FDR-corrected_ = 0.013, *p*_perm,FDR-corrected_ = 0.011) (Fig. 3C).

In contrast to mean DMN connectivity, mean sample entropy within the DMN, which proposes spontaneous brain activity of the DMN indicating qualities of the subjective experience, did not show significant changes after the administration of psilocybin (*F*_1,19_ = 0.578, *p* = 0.456, *p*_perm_ = 0.460, Supplementary Fig. S1). However, more detailed investigations of the DMN subregions could demonstrate a significant psilocybin-induced rise in entropy for TempA (*F*_1,19_ = 9.654, *p*_uncorrected_ = 0.006, *p*_perm,uncorrected_ = 0.006), Aud1 (*F*_1,19_ = 5.612, *p*_uncorrected_ = 0.029, *p*_perm,uncorrected_ = 0.027), and Aud2 (*F*_1,19_ = 4.561, *p*_uncorrected_ = 0.046, *p*_perm,uncorrected_ = 0.046), while the other DMN regions did not reveal significant changes (*p*_uncorrected_ > 0.05 and *p*_perm,uncorrected_ > 0.05, Fig. 3B, C). Alterations of entropy and within-DMN strength in TempA, Aud1, and Aud2 did not correlate to each other.

### Strong association between relapse intensity and psilocybin-induced changes of GBC and DMN connectivity in the ADE group

**Fig. 4:**
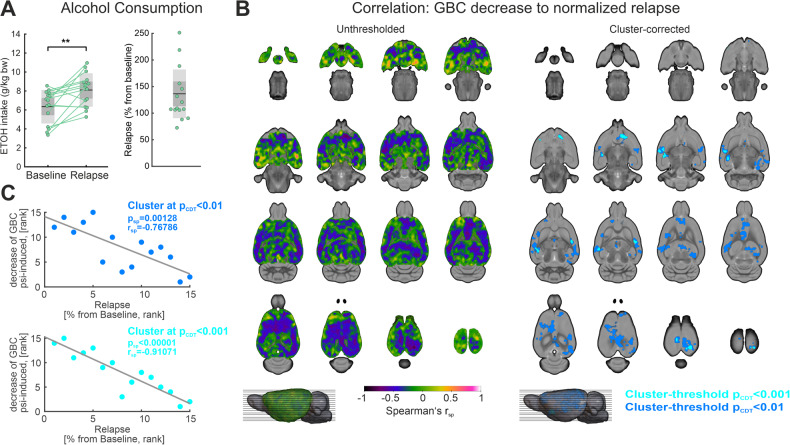
Correlations of global brain connectivity (GBC) decrease to alcohol relapse intensity in the ADE group. **A** Alcohol consumption was significantly higher during relapse when compared to baseline (*p* < 0.01; average ETOH intake at baseline (mean ± SD): 6.35 ± 1.75 g/kg bodyweight; average ETOH intake at relapse (mean ± SD): 8.10 ± 1.74 g/kg bodyweight). The right panel illustrates the percentual change of alcohol consumption (from baseline) during relapse. **B** The left panel shows an unthresholded map of correlation coefficients (Spearman’s r_sp_), which are voxel-wise calculated between the GBC decrease of the 15 ADE rats and their normalized relapse intensities. Areas of strongly negative correlation coefficients are illustrated in blue to violet. The right panel demonstrates the regions surviving cluster correction (*p*_FWE cluster-corrected_ < 0.05; cluster-defining threshold *t* > 2.65 corresponding to *p*_CDT_ < 0.01, dark blue; cluster-defining threshold *t* > 3.85 corresponding to *p*_CDT_ < 0.001, light blue), covering predominantly posterior and anterior cortical regions of the DMN, somatosensory areas and the ventral striatum. Correlation coefficients were transformed to t-values prior to cluster-correction. **C** Scatter plot illustrating the association between relapse intensity and mean decrease of GBC in the respective clusters (*p*_CDT_ < 0.01, dark blue, *p*_CDT_ < 0.001, light blue). ADE alcohol deprivation effect rats (*n* = 15); CDT cluster defining threshold, ETOH ethanol, FC functional connectivity, Psi psilocybin, Sal saline, SD standard deviation, **p < 0.01. For abbreviation of brain regions, see Fig. 2. Prior to calculation of correlation coefficients, connectivity metrics for psilocybin and saline were first compared to its respective baseline, and then subtracted from each other, resulting in ΔSal-Psi values which reflect the decrease of FC.

To analyze psilocybin-induced effects on brain function in alcohol-dependent rats, we first used a categorical approach to distinguish treatment-by-subgroup interaction effects within our repeated measures ANOVA design. Treatment-by-subgroup interaction analysis revealed no significant differences in GBC or NBS for the ADE group upon psilocybin administration when compared to controls (Supplementary Fig. S2). In line with this, subgroup-specific NBS and GBC investigations revealed relatively comparable patterns of psilocybin-induced hypoconnectivity on a whole-brain level in both ADE and control animals (Supplementary Fig. S3). While the categorical discrimination between ADE and control animals in the repeated measures ANOVA design allows for a dichotomous investigation of different psilocybin-induced effects between the two subgroups, more subtle effects of psilocybin may be seen when taking the intensity of the ADE phenotype into consideration. For that, we characterized the phenotype of ADE animals by their relapse intensity (Fig. 4A), which is calculated as the percentage change of alcohol consumption during relapse in comparison to baseline. We did not include baseline drinking rates as covariate into the analysis as baseline drinking rates were not significantly correlated to mean DMN connectivity changes, within-DMN strength alterations in any of the 12 DMN regions, and GBC changes (Supplementary Fig. S4A–C), supporting the use of relapse intensity as drinking measure of the ADE phenotype.

First, investigating Spearman’s rank correlation between GBC and relapse intensity in ADE rats on a whole-brain level, we found a significant association between the normalized relapse rates and the psilocybin-induced decrease of GBC in a broad cortical and hippocampal cluster (Fig. 4B, *p*_FWE cluster-corrected_ < 0.05, cluster-defining threshold *t* > 2.65 corresponding to *p* < 0.01, dark blue, and *t* > 3.85 corresponding to *p* < 0.001, light blue). Specifically, while low relapse intensity in ADE rats led to a relatively strong psilocybin-induced decrease of GBC in these clusters, this effect was less pronounced in ADE rats with a strong ADE phenotype, i.e., high relapse intensity. The effect is reflected by the negative slope of the correlation between relapse intensity and psilocybin-induced GBC decrease illustrated in Fig. 4C. The discovered cluster demonstrated close similarity to the within-subject cluster of psilocybin-induced GBC decrease in all rats (Fig. 2A), covering mostly prefrontal, postero-parietal and hippocampal regions. Interestingly, the association was strongest in parietal auditory and visual areas and in the nucleus accumbens.

**Fig. 5:**
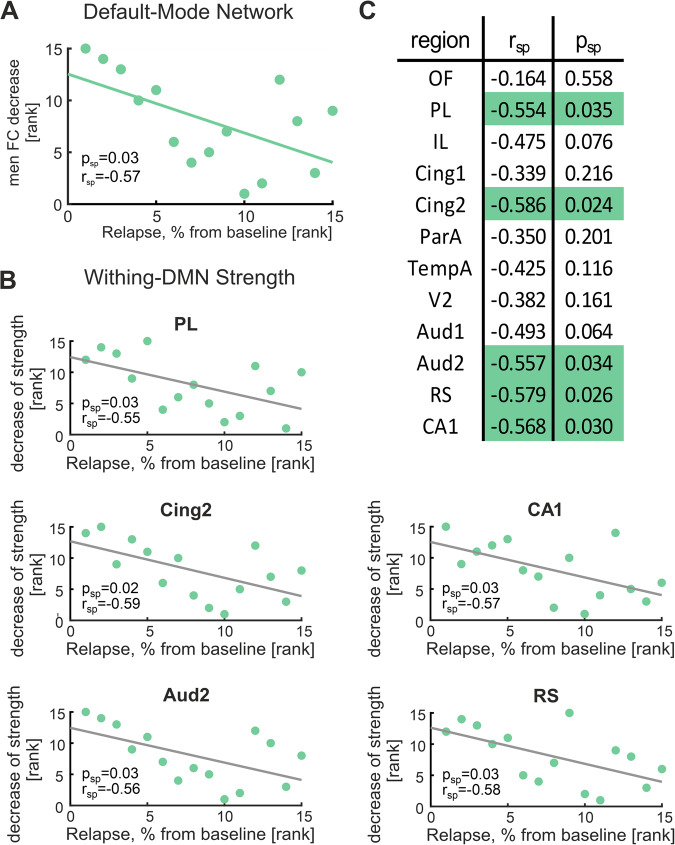
Correlations of within-DMN connectivity decrease to alcohol relapse intensity in the ADE group. **A** Psilocybin-induced decrease of mean DMN functional connectivity significantly correlated to the normalized relapse rate (Spearman’s *r*_sp_ = −0.57, *p*_sp_ = 0.05). **B** Further analyses showed significant correlations between the decrease of within-DMN strength in five DMN regions (PL, Cing2, Aud2, CA1, RS) and relapse intensity in the ADE group. **C** Illustrates the correlation coefficients (Spearman’s r_sp_) and *p*-values (*p*_sp_) between within-DMN strength and relapse intensity for all 12 DMN regions. Significant values are marked in green. ADE alcohol deprivation effect rats (*n* = 15), DMN default mode network, ETOH ethanol, FC functional connectivity, Psi psilocybin, Sal saline, SD standard deviation, **p < 0.01. For abbreviation of brain regions, see Fig. 2. Prior to calculation of correlation coefficients, connectivity metrics for psilocybin and saline were first compared to its respective baseline, and then subtracted from each other, resulting in ΔSal-Psi values which reflect the decrease of FC.

Moreover, Spearman’s rank correlation showed a significant association between the normalized relapse rates and the psilocybin-induced mean FC decrease in the DMN (Spearman’s *r*_sp_ = −0.57, *p* = 0.03, Fig. 5A). This implies that the general effect of psilocybin-induced decrease of DMN connectivity was dependent on the alcohol relapse phenotype: While ADE rats with low relapse intensity showed a strong decrease of DMN connectivity upon psilocybin administration, comparable to the control rats, DMN connectivity decrease was less pronounced in ADE rats with strong relapse intensity. When dissecting this association for subregions of the DMN using local within-DMN strength, a comparable relationship could be detected for the two prefrontal regions PL (*r*_sp_ = −0.55, *p*_sp,uncorrected_ = 0.03) and Cing2 (*r*_sp_ = −0.59, *p*_sp,uncorrected_ = 0.02), Aud2 (*r*_sp_ = −0.56, *p*_sp,uncorrected_ = 0.03), RS (*r*_sp_ = −0.58, *p*_sp,uncorrected_ = 0.03), and CA1 (*r*_sp_ = −0.57, *p*_sp,uncorrected_ = 0.03), suggesting these regions as drivers of the phenomenon (Fig. 5B).

## Discussion

Our randomized, controlled crossover pharmaco-fMRI study on psilocybin effects in healthy and alcohol-dependent rats demonstrates brain-wide changes in functional connectivity and whole-brain network organization. Importantly, while subgroup-specific effects in alcohol-dependent rats were absent, a clear association between alcohol relapse intensity and the connectivity changes on a whole-brain and DMN level could be demonstrated.

Firstly, we detected a highly significant psilocybin-induced decrease of GBC within a wide-spread cortical cluster. In line with this finding, ROI-based NBS analysis showed a decrease of FC upon psilocybin administration in a broad cortical network, which was accompanied by a well-localized hub-region specific increase of connectivity from the DRN, the serotonergic core region of the brain, and the hypothalamus. Regional graph metrics corroborated this bi-directional pattern of network alterations, while global graph metrics additionally suggested an increase of small-world organization and whole-brain integration as neural mechanisms of psilocybin. Further, we could provide translational evidence for the proposed phenotype of DMN hypoconnectivity following acute psilocybin application in humans as well as demonstrating increased sample entropy for certain DMN regions. Lastly, although whole-brain network alterations upon psilocybin administration were mostly comparable between alcohol-dependent and control rats, we could demonstrate a modulation of voxel-wise GBC and DMN connectivity by alcohol relapse intensity.

With this study, we could replicate several psilocybin-induced network effects reported in human MRI studies in the rat brain. This is an important translational finding and crucial for the preclinical psychedelic research field, as it provides for the first time construct validity and, thus, supports the use of animal models to assess the efficacy and mode of action of psychedelic substances in psychiatric disorders. Moreover, we show that psilocybin-induced effects of decreased global brain connectivity and DMN hypoconnectivity are inversely correlated to the intensity of alcohol relapse, where stronger relapse is associated with weaker psilocybin effects. This finding has clinical implications, suggesting that a standard therapeutic dose of psilocybin may only be effective in less severe AUD cases, and that higher doses are probably required for severe AUD cases.

### Psilocybin acutely decreases broad cortical functional connectivity

Our multidimensional FC analyses provide converging evidence for an acute psilocybin-induced decrease of FC in the cortex, replicating analogous findings in human studies for the first time in rats [14, 16, 39]. GBC analysis corroborated recent results based on comparable preprocessing without global signal regression from Preller et al. (see Supplementary Fig. S4A from ref. [39]) by demonstrating a pattern of psilocybin-induced cortical and hippocampal GBC decrease. Specifying this finding with NBS, we could show that decreased ROI-to-ROI connections were mostly located between prefrontal, posterior-parietal and associative brain regions. Studies in both animals and humans provide evidence for this phenotype of cortical brain hypoconnectivity [14–19]. Thus, a translational EEG study in rats showed that psychedelic substances induced an overall global decrease of cortical functional connectivity [58]. In line with the results from our study, Carhart-Harris et al. [16] detected decreased neuronal activity in the anterior cingulate and medial prefrontal cortices (mPFC) in humans with fMRI, leading to diminished FC between the mPFC and the posterior cingulate cortex. The group hypothesized that the effects of psychedelics are caused by a 5-HT2A receptor-induced dysregulation of spontaneous population-level neuronal activity that leads to a temporary network desynchronization [59]. A general decrease in directed FC broadly across the cortex could also be found using MEG under acute psilocybin treatment [60], albeit their results for undirected FC were less strong. While again Carhart-Harris et al. [15] found an LSD-induced decrease of rs-FC between retrosplenium and hippocampus, which they put in context of ego dissolution, the group described strongly increased visual cortex connectivity at the same time, in contrast to our study. Other studies also describe increased FC or bi-directional cortical FC alterations, although predominantly during task-performance [61]. Despite such heterogeneity in fMRI findings (for review see ref. [12]), the most consistently replicated pattern is a psychedelic-induced acute decrease of FC in the human DMN [14–19]. Importantly, when focusing on the DMN in our study [47], we could provide translational evidence for exactly this key finding for the first time in rats. DMN hypoconnectivity was associated with a strong decrease of within-DMN FC strength for almost all DMN regions. Of note, DMN alterations could not only be observed in FC measures but were also present in single-ROI sample entropy analysis of TempA and auditory cortices, corroborating psychedelic effects on brain entropy in humans [62].

### Psilocybin specifically increases FC in a subnetwork with the hub-regions DRN and hypothalamus

In addition to the hypoconnectivity within the delineated wide-spread cortical network, our ROI-based NBS approach revealed a well-localized psilocybin-induced increase of FC in circuits focusing on subcortical brain structures. Specifically, DRN and hypothalamus acted as hub regions of these hyperconnected circuits, with increased FC to various cortical areas including prefrontal, sensory-processing and retrosplenial regions. With psilocybin’s mode of action as an agonist on 5-HT receptors (reviewed by Ling et al. [63]), its prominent effect on DRN connectivity should be seen in the context of the DRN’s role as the major serotonergic nucleus in the brain [64, 65]. Raphe 5-HT neurons orchestrate cortical reorganization among different sensory and effector systems via modification of trans-synaptic signaling [64]. Grandjean et al. [30] observed an increase of FC within the 5-HT system and its projection fields upon acute psilocybin administration in a rsfMRI study with mice. However, the group could not demonstrate an increase of FC specifically from the DRN to other cortical regions [30], potentially due to smaller brain sizes and different anesthetic regimens. The psilocybin-induced increases of FC between the DRN and various cortical areas in our study correspond to the anatomical afferent and efferent connections of the DRN (reviewed by Lesch et al. [64, 66]), potentially reflecting the effect of psilocybin treatment on DRN’s modulation of circuit configuration in prefrontal and sensory cortical areas via altered serotonergic signaling [64, 67]. Hence, altered DRN connectivity may be one of the acute processes underlying increased neuroplasticity, which has recently been demonstrated after psilocybin treatment in cortical brain regions [68]. Importantly, our data from graph analysis and GBC could corroborate the idea of altered DRN function underlying psilocybin’s neural mechanism, showing not only a psilocybin-induced increase in local strength and GBC, but also a significantly higher participation index in the DRN. The latter indicates an altered role of the DRN as an intermediate hub between different brain subnetworks upon psilocybin administration, potentially driving increased network integration observed after psilocybin treatment in depressive patients [69]. Altogether, our study aligns with current preclinical evidence highlighting the important role of DRN-mediated circuit effects in the neural mechanisms of psychedelics [70]. In this regard, the current absence of human studies on psilocybin-induced DRN modifications is highly critical, as they are urgently needed to establish its translation validity. Interestingly, we could demonstrate similar FC alterations also for the hypothalamus. The increased FC of the hypothalamus to several cortical regions and its altered hub function may hint at potential neuroendocrine effects of psilocybin as one possible mechanism (reviewed by Schindler et al. [71]). In summary, our data suggest that both regions may be crucial for the process of integration during the psychedelic experience [69].

### Psilocybin acutely increases small-world brain network structure and whole-brain integration

Further corroborating the delineated restructuring of the functional network reported in our study, global graph metrics demonstrated a significant increase in small-world structure after psilocybin administration, which was predominantly driven by optimized brain network integration (ΔL). Small-worldness characterizes the optimal organization present in the healthy brain, providing an ideal balance between regional and long-distance information flow [46]. In correspondence with our findings, Luppi et al. [72]. reported an increase of small-worldness upon acute LSD administration in both segregated and integrated dynamic sub-states in a human fMRI study. However, in contrast to our results, changes in small-worldness were in a large part driven by optimized network segregation (ΔC), while the influence of network integration (ΔL) was opposing. The selection of distinct sub-states and the different psychedelic substances might contribute to such diverging effects of psychedelics on ΔL. Importantly, the feature of increased global integration in the brain after psilocybin treatment, as suggested by the change in ΔL in our study, has recently been demonstrated in a randomized-control fMRI study in depressive patients [69], proposing it as a potential underlying brain mechanism for its antidepressant properties.

### Modulation of GBC and DMN connectivity by the alcohol relapse phenotype

While such psilocybin-specific therapeutic mechanisms are increasingly investigated for major depressive disorder, studies focusing on specific mechanistic pathways in AUD are lacking. Investigating psilocybin-induced neural effects in a translational AUD model, our study demonstrates rather comparable effects of psilocybin on FC in alcohol-dependent and control animals, with no treatment-by-subgroup interaction effects in the categorical approach. This is in line with previous investigations using microarray gene chips, exhibiting a limited number of significantly differentially expressed genes in the ADE model compared to control animals [73]. More importantly, our dimensional analyses revealed a clear association between the ADE phenotype and the previously delineated psilocybin-induced FC alterations, providing converging evidence for a modulation of acute psilocybin effects by the intensity of the alcohol relapse.

Firstly, ADE animals with higher relapse intensity experienced weaker effects of psilocybin on GBC, mostly affecting parietal auditory, visual areas and nucleus accumbens. Such diminished psilocybin responsivity can potentially be attributed to a disease-specific anaplasticity phenomenon. The anaplasticity concept is characterized by the loss of neuronal and molecular plasticity following external stimulation in the addicted brain, specifically within the nucleus accumbens [74]. Synaptic plasticity allows for flexibility in behavior. However, when plasticity is impaired, the behavior may become more inflexible, leading to a loss of control of the alcohol consumption. Since animals with a stronger relapse are more prone to develop an addiction including a compulsive, harmful use habit [22, 75], it is likely that the brains of these rats show forms of anaplasticity and thus fail to respond to the applied psilocybin dose as an external trigger adequately.

Secondly, we show that psilocybin-induced hypoconnectivity of the DMN was broadly and significantly correlated with the relapse intensity of alcohol-dependent rats. We could demonstrate that increased relapse intensity correlates with a diminished effect of psilocybin dose on DMN hypoconnectivity, especially of the mPFC, but also of retrosplenial, auditory and hippocampal areas. Excessive alcohol intake has profound structural and functional impact on the brain. Recent MRI experiments in various rodent models of AUD found altered activity and connectivity patterns in numerous brain regions but particularly in the prefrontal cortex [21, 76, 77]. Furthermore, chronic alcohol ingestion causes remodeling of prefronto-cortical neurons, including increased spine density, altered functional connectivity, and impaired cognitive abilities in humans and in rodents [31, 78–81]. Adding on to that, we recently found disrupted mPFC function resulting from the inability to properly regulate glutamate release due to a loss of metabotropic glutamate receptors 2 (mGluR2) in alcohol-dependent rats [82]. These findings are in line with the observation that prefrontal mGluR2/3-mediated long-term depression is specifically suppressed in addicted rats [83], linking the above mentioned anaplasticity concept to a common molecular mechanism of drug addiction. Collectively, these observations are of particular importance, since it has been previously demonstrated that psilocybin’s primary target, the serotonin 5-HT_2A_ receptor, and the mGluR2 can assemble into a functional complex and modulate each other’s function [84–88]. This heteromeric complex has been implicated in the mechanism of action of psychedelics, and its role has been demonstrated in several in vivo systems [89–93]. Therefore, it is likely that aberrant prefronto-cortical mGluR2 function contributes to the observed blunted psilocybin-induced DMN response in alcohol-dependent rats.

Considering that psilocybin-induced changes of DMN-network integrity correlate with the psychedelic experience [94] and that the psychedelic experience has been shown to mediate the effect of psilocybin dose on therapeutic outcomes (e.g. ref. [3]), our translational neuroimaging data suggest that patients with AUD need an individual adaptation of the psilocybin dosage for a successful treatment. In accordance with the modulation of the psilocybin effect by the alcohol relapse intensity, we argue that the psilocybin dosage needs an adaptation with regard to the individual alcohol consumption level. Corresponding with this hypothesis, Maclean et al. [95] already proposed in 1961 that “small-dose techniques are less effective as they do not lead to a full realization of the therapeutic potential of the experience” in an LSD-trial on AUD. Only future clinical trials that take the here obtained findings into consideration will be able to answer this question and demonstrate the efficacy of psychedelics as a new treatment option for AUD.

### Limitations

The small sample size of the control group limits the power of detecting treatment-by-subgroup interaction effects. Thus, more subtle differences of psilocybin-induced effects between ADE and control animals might potentially be undetected. Taking into account the broad distribution of the ADE phenotype and its clear association to the neuronal effects of psilocybin demonstrated in our study, future studies are needed to delineate how the intensity of the ADE phenotype exactly blunts the effects of psilocybin. Importantly, the unequal sample sizes did not affect our statistical analyses. To control basic assumptions of the repeated measures ANOVA, we integrated Shapiro–Wilk [53] and Bartlett’s test [54] to examine normal distribution and equality of variance for the reported metrics. Additionally, we incorporated non-parametric permutation tests for data with non-normal distribution.

To rule out possible confounders of the study, such as alcohol-induced changes in tolerance regarding anesthesia or alterations of 5-HT receptor expression in ADE rats, several control analyses and experiments were performed. To exclude cross-tolerance effects between ADE rats and anesthesia during scanning, we conducted a baseline comparison between ADE and control rats at the first time point of measurement (Supplementary Fig. S5). No significant differences could be observed in rsfMRI parameters (GBC and NBS). Similarly, 5-HT_2A_ receptor levels were investigated between ADE rats and age-matched controls. No expression differences were found between groups (Supplementary Fig. S6), suggesting that the observed network differences in the study are a result of system level adaptations in the ADE model. The use of anesthesia is a major difference in comparison to human fMRI studies. However, the established protocol involving the combination of light anesthetics is widely regarded as the gold standard for the assessment of resting-state networks in rats, showing a good correspondence to brain networks of awake rats, and simultaneously reducing the side-effects of awake rodent imaging such as stress [33, 96]. In this context, it is remarkable that we observed analogous patterns in both rats and humans, even in the presence of potential confounding factors related to anesthesia. Nevertheless, functional data obtained from awake rodents would be useful to confirm the presented findings.

We did not apply global signal regression (GSR) in our rsfMRI analysis due to several inherent caveats of this technique: GSR is a method originally developed to account for potential sources of physiological noise [97]. However, it has been shown to artificially introduce negative correlations [97] and applies its correction in a region-unspecific way. Preferably, we used an advanced regression method [35] that deploys a region-specific correction for physiological noise (see Supplementary Fig. S7B), taking into account that the brain is inhomogeneously affected by cardiorespiratory activity. Regions with a large proportion of variance of the fMRI signal explained by cardiac pulsation and respiratory cycle were predominantly located in close proximity to large blood vessels (e.g., ventral and posterior-dorsal brain areas, see Supplementary Fig. S7B), while cortical regions demonstrating main psilocybin treatment effects (e.g., IL and PL) were only marginally affected by the cardiorespiratory signal. Such correction is necessary as first, psilocybin influenced heart and respiration rate differently in comparison to saline (see Supplementary Fig. S7C, drug effect on heart rate: *F*_1,19_ = 8.082, *p* = 0.010; *p*_perm_ = 0.001, drug effect on respiration rate: *F*_1,19_ = 6.611, *p* = 0.019, *p*_perm_ = 0.019) and second, ADE animals demonstrated lower mean heart and respiration rates at baseline (see Supplementary Fig. S7D, heart rate: *p*_perm_ = 0.0187, respiration rate: *p*_perm_ = 0.0014). Comparable effects of chronic alcohol exposure on heart and respiration rates have been described in various animal models [98, 99]. Of note, Aztec removes confounds at single animal level, not only considering differences in mean heart and respiration rates but also filtering the different temporal variations in heart and respiratory cycles (e.g., heart rate variation, respiratory volume, etc.). Additionally, we regressed out the mean CSF signal, potentially reflecting physiological noise including heart rate and respiration rates and applied band pass filtering (0.01–0.1 Hz). Band pass filtering focusses the analyses on the range of interest, ruling out strong effects from heart and respiration rate, which are normally fluctuating between 0.75 HZ (~45 bpm, respiration rate) and 3.5 Hz (~210 bpm, heart rate). Subsequent correlation analyses at baseline before substance administration could corroborate the effectiveness of our approach by not finding significant influences of mean heart and respiration rates on our rsfMRI measurements for GBC and functional connectivity (Supplementary Fig. S8).

### Supplementary information

SUPPLEMENTAL MATERIAL

## Data Availability

All data needed to evaluate the conclusions in the paper are present in the paper and/or the Supplementary Materials.

## Code availability

Codes used for graph analysis and NBS are freely available from the Brain Connectivity Toolbox [44]. In-house MATLAB codes for FC, entropy, correlation analysis and illustration of results are available upon request.
