# Supplementary material for: Psilocybin-induced default mode network hypoconnectivity is blunted in alcohol-dependent rats
Source: Transl Psychiatry. 2023 Dec 14;13:392. doi: 10.1038/s41398-023-02690-1 (PMC10721862; doi:10.1038/s41398-023-02690-1)
Supplement: Supplementary file 1 — SUPPLEMENTAL MATERIAL [file 41398_2023_2690_MOESM1_ESM.pdf]

SUPPLEMENT

MRI acquisition

The MRI experiments were carried out at a small-animal 9.4 Tesla MRI scanner (Bruker BioSpec, Ettlingen, Germany) with Avance III hardware, BGA12S gradient system (maximum strength 705 mT/m) and Paravision 6 software. The cryocoil consists of an anatomically shaped four-channel receive-only low temperature array combined with quadrature whole-body volume transmitter operating at room temperature. The rats were initially anesthetized with 4% isoflurane (Baxter Deutschland GmbH, Unterschleissheim, Germany) in a mixture of 70% N2 and 30% O2. After positioning in the scanner, isoflurane level was reduced to 2.5%, and medetomidine (Domitor, Janssen-Cilag, Neuss) was injected as a bolus (0.03 mg/kg, s.c.). The isoflurane level was reduced to 0.5% during acquisition of the structural image and the animals received continuous medetomidine (0.06 mg/kg/h). We monitored sedation depth throughout the experiment via recording the respiratory and cardiac parameters at 10-ms resolution using a signal breakout module (Small Animal Instruments Inc., NY, USA) and a 4-channel recorder (Velleman® N.V., Gavere, Belgium). Body temperature was maintained at 37°C throughout the session.

Graph theoretical analysis

Strength (sum of connections for a given node), local clustering coefficient (number of connections between nodal neighbors, normalized to the maximum number of possible connections) and participation index (ratio of intermodule strength to the total nodal strength) were assessed to explore a specific region's role in the network structure.

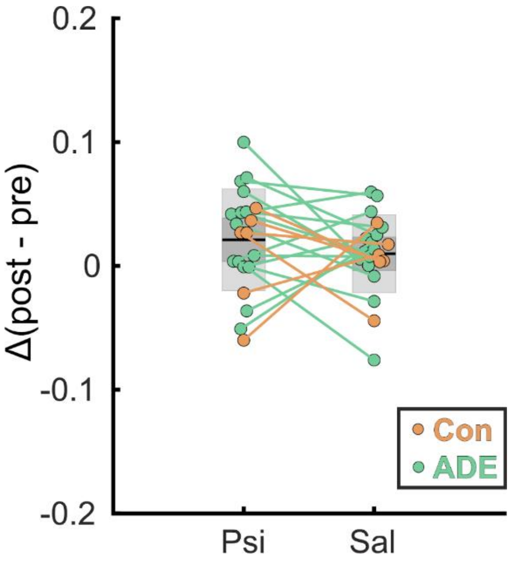

Fig. S1: Mean sample entropy of the default mode network (DMN). Psilocybin administration did not significantly affect mean DMN sample entropy compared to placebo ($F_{1,19}$= 0.58, p>0.05). For mean entropy calculation, all voxels of the DMN as defined in the main part of the manuscript were used.

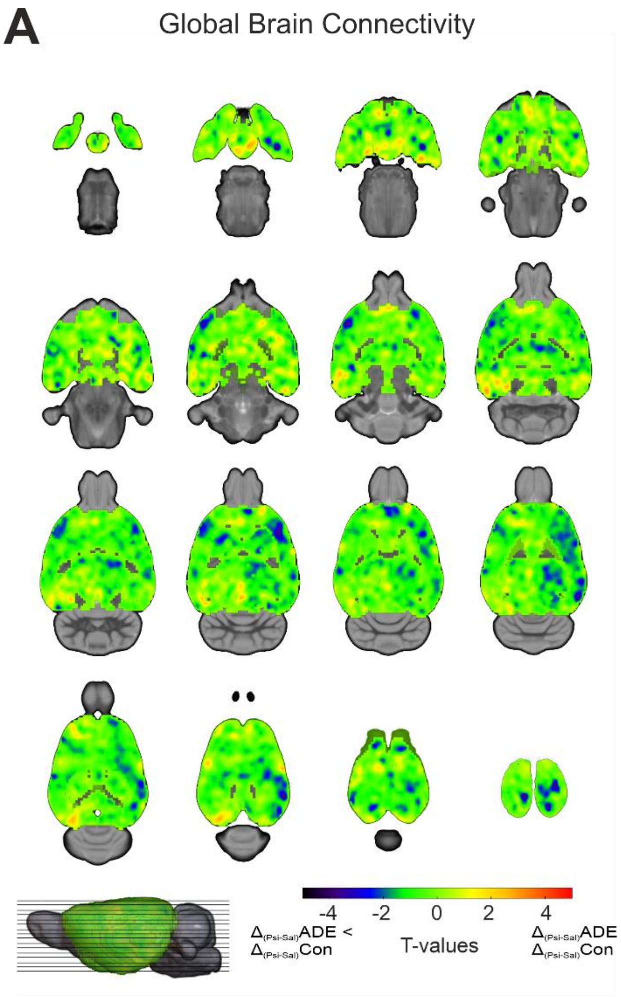

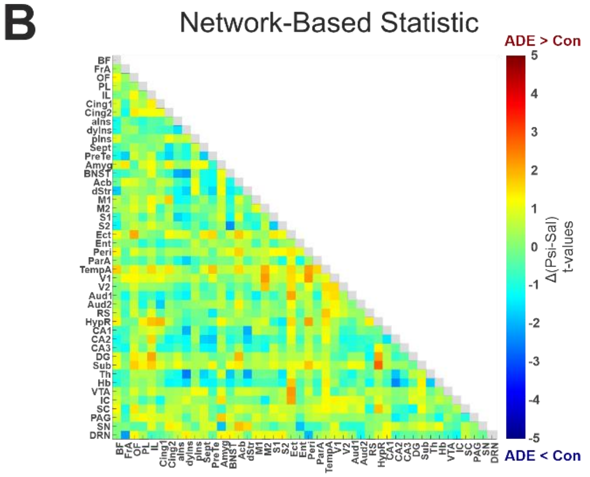

C Graph Analysis

Global Metric	Interaction $F_{(1,19)}$
SWP	1.23
Δ_L	1.09
Δ_C	0.60

Region	Strength	Partic. Ind.
	Interaction	
	$F_{(1,19)}$	$F_{(1,19)}$
PL	0.16	0.00
Ect	0.99	0.71
ParA	0.15	0.31
TempA	2.90	1.15
V1	1.18	0.36
V2	0.67	0.70
Aud1	0.17	0.36
Aud2	0.09	0.06
RS	0.01	0.22
HypR	0.05	0.29
Sub	0.85	0.43
DRN	3.55#	1.42

D Default-Mode Network

Region	OF	PL	IL	Cing1	Cing2	ParA	TempA	V2	Aud1	Aud2	RS	CA1
Within-DMN Strength Interaction, F(1,19)	0.79	0.04	1.27	0.09	0.53	0.00	3.24#	0.20	0.16	0.00	0.39	0.06
Entropy Interaction, F(1,19)	2.09	0.05	0.00	0.17	0.01	0.01	0.00	0.01	0.04	0.00	0.12	0.44

Fig. S2: Subgroup-by-treatment interaction effects for global brain connectivity (GBC), network-based statistic (NBS), graph analysis, within-default-mode-network (DMN) strength and entropy within the DMN. (A) Subgroup-by-treatment interaction effect 18 minutes after psilocybin administration, comparing psilocybin effects in ADE to control animals. Of note, no clusters survived a cluster-correction ($p_{FWE\ cluster\text{-}corrected}>0.05$) with a cluster-defining threshold of $t>2.54$ (corresponding to $p<0.01$) and $t>3.58$ (corresponding to $p<0.001$). Psilocybin-induced GBC decrease was slightly more pronounced in the ADE group compared to the control animals in blueish regions. **(B)** NBS, **(C)** global and local graph metrics, **(D)** within-DMN strength and entropy of the DMN regions did not reveal any significant subgroup-by-treatment interaction effect ($p>0.05$).

ADE, alcohol deprivation effect rats (n=15); ANOVA, analysis of variance; Con, control rats (n=6); Δ_C, deviation of the clustering coefficient from both lattice and random networks constructed with the same number of nodes and the same degree distribution; Δ_L, deviation of the network's characteristic path length from both lattice and random networks constructed with the same number of nodes and the same degree distribution; Psi, psilocybin; Sal, saline; # $p<0.10$; For abbreviation of brain regions, see Fig. 2. All psilocybin and saline values were first compared to its respective baseline, before feeding the difference into the repeated measures ANOVA.

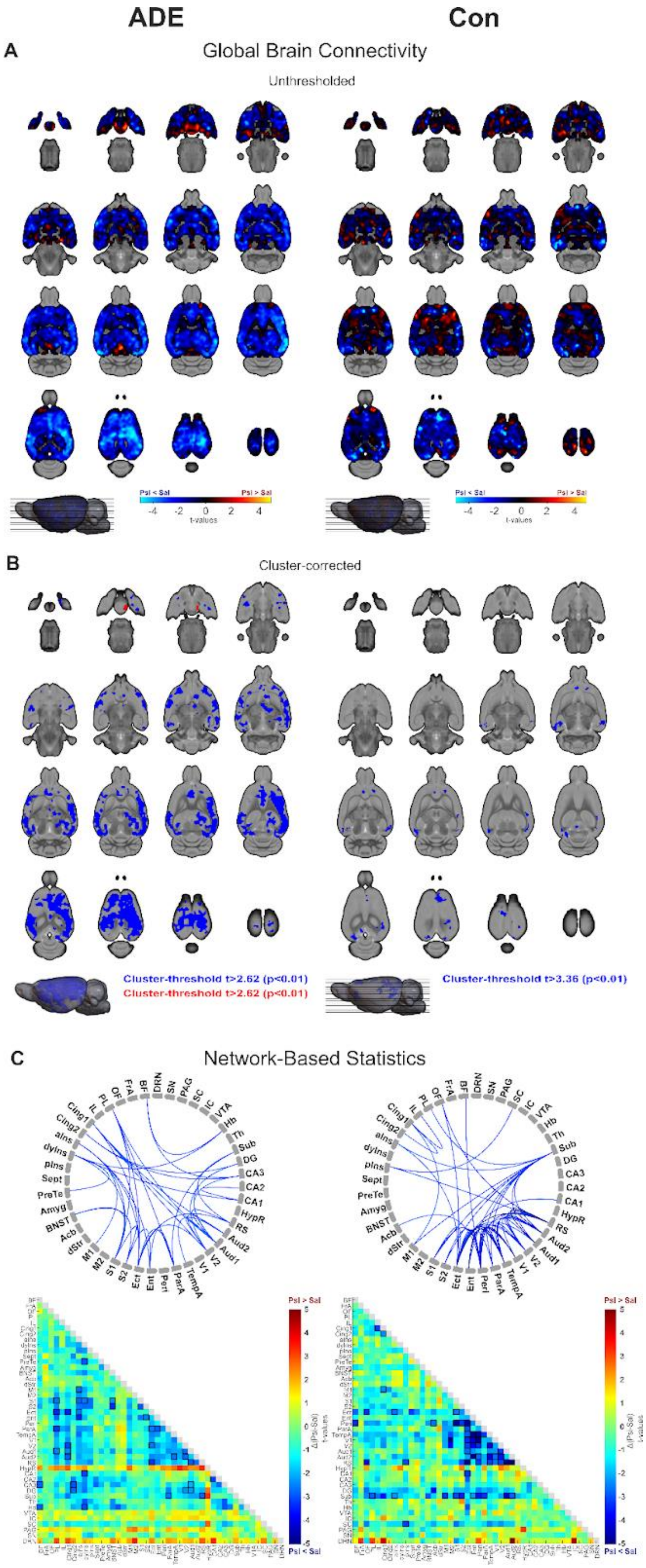
ADE
Con
A
Global Brain Connectivity
Unthresholded
Psi < Sal
Psi > Sal
-4 -2 0 2 4
t-values
B
Cluster-corrected
Cluster-threshold t>2.62 (p<0.01)
Cluster-threshold t>2.62 (p<0.01)
Cluster-threshold t>3.36 (p<0.01)
C
Network-Based Statistics
IL PL OF FrA BF DRN SN PAG SC IC VTA Hb Th Sub DG CA3 CA2 CA1 HypR RS Aud2 Aud1 V2 V1 TempA ParA Peri Ent Ect S2 S1 M2 M1 dStr Acb BNST Amyg PreTe Sept pIns dyIns aIns Cing2 Cing1
Psi > Sal
Psi < Sal
Δ (Psi-Sal) t-values

Fig. S3: Subgroup-specific psilocybin effects on GBC and FC compared to placebo. (A) Unthresholded t-value map illustrating post-hoc voxel-wise GBC analysis of the within-subject treatment condition comparing psilocybin to placebo in ADE rats, depicted on the left panel. Regions exhibiting reduced GBC under psilocybin are indicated in blue to light blue, while red to yellow indicates regions with increased GBC. Correspondingly, the right panel demonstrates an unthresholded t-value map of GBC comparison using a post-hoc analysis of the within-subject treatment condition comparing psilocybin to the placebo condition in control rats. **(B)** areas surviving cluster-correction ($p_{FWE\ cluster\text{-}corrected}<0.05$) of the respective unthresholded t-value map of panel A, with a cluster-defining threshold of $t>2.62$ (dark blue and red, cluster-size ≥ 6991 voxels, ADE) for the left panel and of $t>3.36$ (dark blue, cluster-size ≥ 82 voxels, Con) for the right panel. **(C)** NBS results (lower panels) comparing FC alterations during psilocybin treatment and placebo demonstrate a comparable but slightly stronger pattern of psilocybin-induced cortical hypoconnectivity (blue) in control animals and ADE rats (left panel for ADE: $p_{NBS}<0.05$; primary threshold $t_{pt}>2.47$, corresponding to $p_{pt}<0.01$; right panel for control animals: $p_{NBS}<0.05$; primary threshold $t_{pt>}2.76$, corresponding to $p_{pt}<0.01$). Data is illustrated in clockwise manner in a connectivity matrix as t-values (psilocybin vs saline) with black boxes marking the connections of the cluster surviving the NBS. The upper panels depict significant t-test results of a test for directionality of the main effect in control animals (upper right panel) and in ADE rats (upper left panel).

A

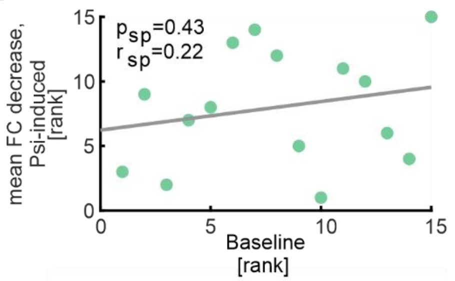

B

DMN region	Relapse (% of baseline consumption)		Baseline (g/kg bw)	
	Correlation coeff. r_{sp} (Spearman's)	p-value	Correlation coeff. r_{sp} (Spearman's)	p-value
**OF**	-0.164	0.558	-0.007	0.985
**PL**	**-0.554**	**0.035**	0.250	0.368
**IL**	-0.475	0.076	0.050	0.863
**Cing1**	-0.339	0.216	-0.054	0.853
**Cing2**	**-0.586**	**0.024**	0.439	0.103
**ParA**	-0.350	0.201	-0.057	0.842
**TempA**	-0.425	0.116	0.214	0.442
**V2**	-0.382	0.161	-0.029	0.923
**Aud1**	-0.493	0.064	0.225	0.419
**Aud2**	**-0.557**	**0.034**	0.257	0.354
**RS**	**-0.579**	**0.026**	0.111	0.695
**CA1**	**-0.568**	**0.030**	0.411	0.130
**mean DMN**	**-0.568**	**0.030**	0.221	0.427

C

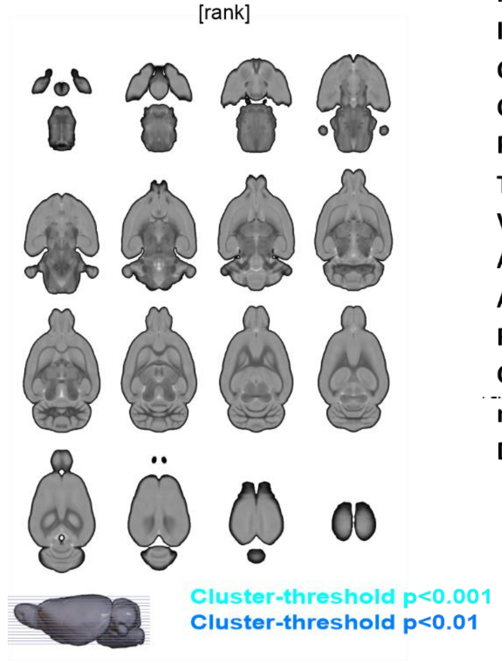

Fig. S4: Correlation between different drinking measures and rsfMRI metrics. The association between baseline drinking rate and the mean FC decrease in the default mode network is illustrated in **(A)**. The table in **(B)** demonstrates Spearman's correlation coefficients and p-values for the association between alcohol consumption during relapse (% of baseline) and baseline (right panel) and the within DMN strength and mean DMN functional connectivity.

Significant correlations are marked in bold ($p_{uncorrected}$<0.05) with green background. Importantly, while several associations between relapse intensity and within DMN strength/mean DMN FC reached significance ($p_{uncorrected}$<0.05 for PL, Cing2, Aud2, RS, CA1, mean DMN FC), no significant associations to baseline drinking rate were found. For **(C)**, voxel-wise correlation coefficients (Spearman's r_{sp}) were calculated between the GBC scores of the 15 ADE rats and their baseline drinking rates before being cluster corrected (same methodological approach as in Fig. 5B of the main part of the manuscript). No cluster survived the correction ($p_{cluster-corrected}$<0.05; cluster-defining threshold t>2.65 (corresponding to p<0.01), dark blue; cluster-defining threshold t>3.85 (corresponding to p<0.001), light blue).
ADE, alcohol deprivation effect rats (n=15); Con, control rats (n=6); GBC, global brain connectivity

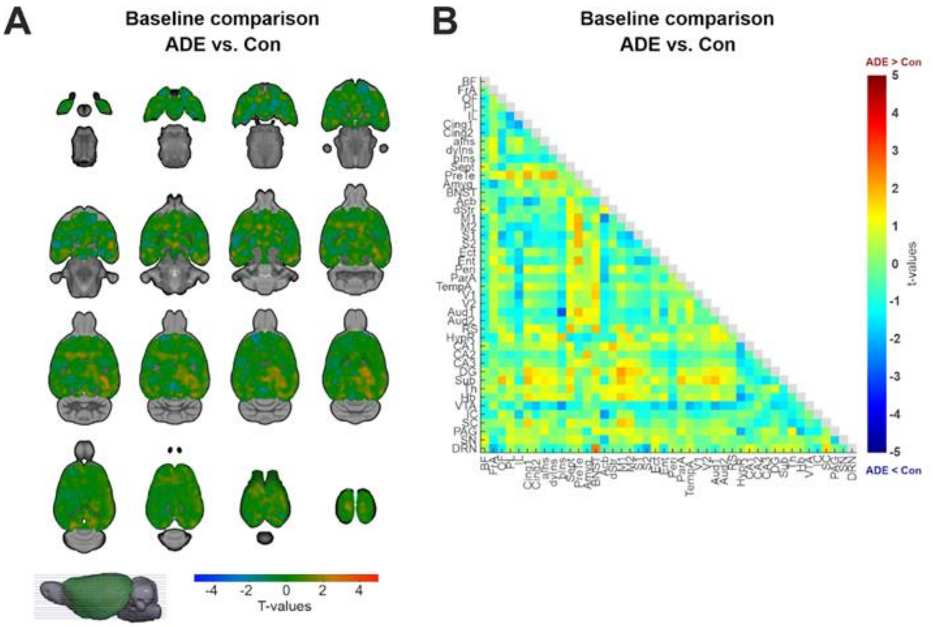

Fig. S5: Baseline comparison between ADE and control rats at first measurement. We performed GBC and NBS analysis of the baseline condition (= pre-injection of the first session, 8.5 minutes) to evaluate baseline differences between ADE and controls without the pharmacological challenge. **(A)** The unthresholded t-value map comparing GBC between ADE and control animals at the time point before injection shows only very weak differences between the two groups (mostly green color) with no cluster surviving cluster-correction even at a lenient cluster defining threshold of t>2.54 (corresponding to p_{CDT}<0.01). Similarly, (**B)** NBS analysis between ADE and control rats demonstrated very weak differences between the two groups at baseline (light blue to light yellow color) with no cluster surviving NBS at a primary cluster defining threshold of p_{pt}<0.01.
ADE, alcohol deprivation effect rats (n=15); Con, control rats (n=6); CDT, cluster-defining threshold; GBC, global brain connectivity, NBS, network-based statistics; pt, primary threshold

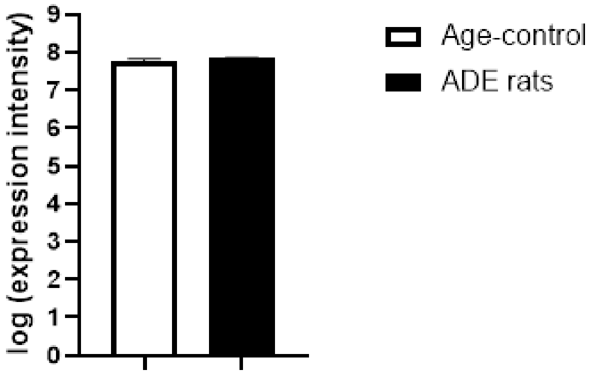

Fig. S6: HTR2A transcript levels show no expression differences between alcohol and control rats. Expression patterns of HTR2A mRNA levels were measured by illumina microarray in the nucleus accumbens and are presented as logarithmic values of the normalized expression intensities. Two-sample t-test between the two groups did not reveal significant differences (n=6 animals per group, $p>0.05$, unpublished data).
ADE, alcohol deprivation effect rats, HTR2A, 5-hydroxytryptamine receptor 2A

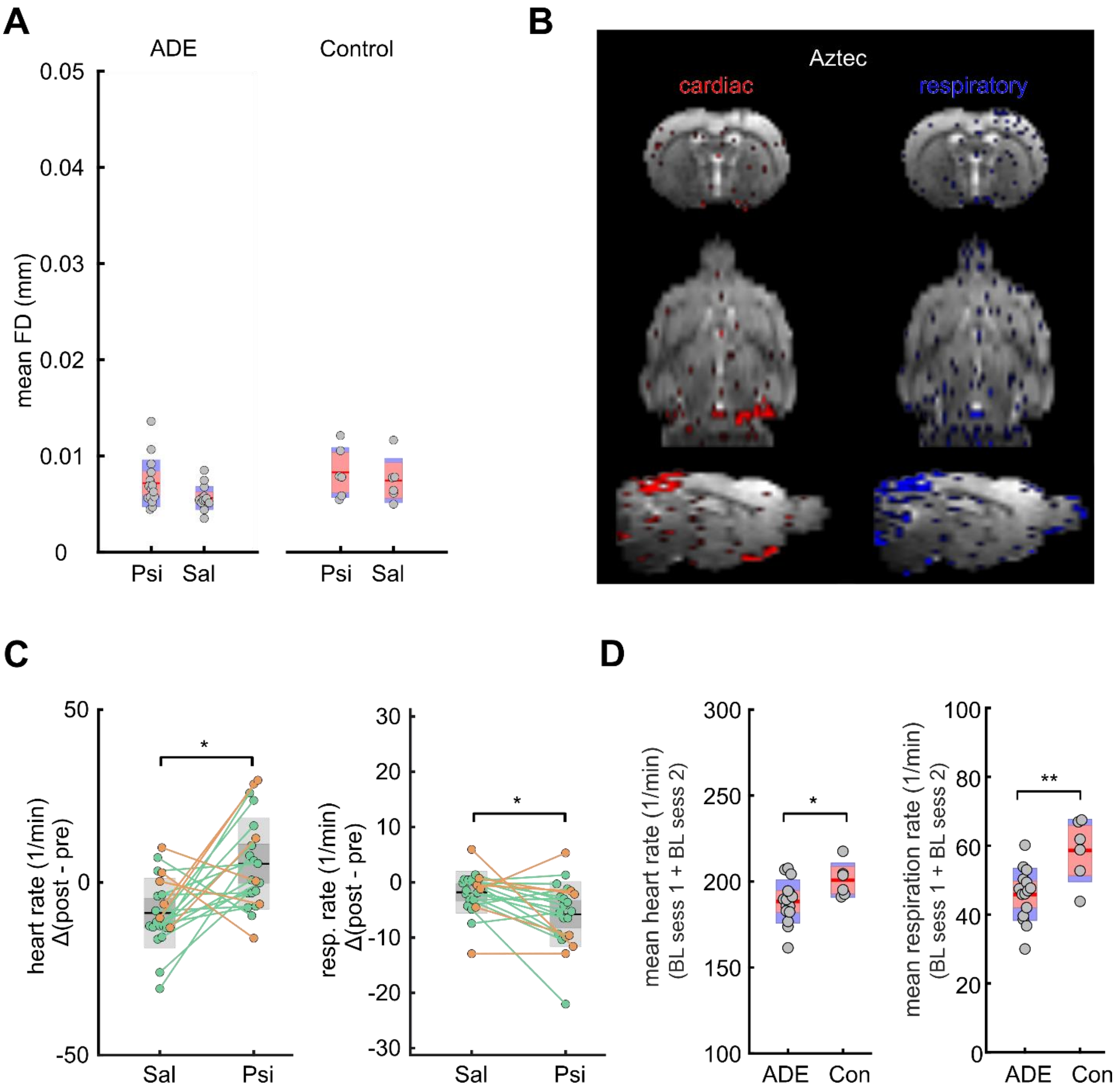

Fig. S7: Assessment and correction for motion, heart and respiration rate. (A) Analysis of average framewise displacement (FD) indicates a generally very low level of motion during the fMRI experiment (mean FD<<0.05 mm). No significant psilocybin treatment effects could be found in the ADE and control group, respectively (post-hoc paired t-test p>0.05 between psilocybin and saline condition). Further, no significant treatment-by-subgroup interaction effect could be demonstrated (unpaired t-test comparing FD_{Psi}-FD_{Sal} between ADE and control condition, p>0.05). **(B)** Exemplary illustration of the region-specific correction for physiological noise (cardiac and respiratory signal) applied by Aztec software [78]. Of note, this correction method particularly takes into account that the brain is non-homogeneously affected by cardiorespiratory activity. Bright red and blue colors depict areas with relatively strong correction for physiological noise. **(C)** Comparison of heart rate differences (Δpost-pre-injection, left panel) between psilocybin and saline treatment demonstrated a significant drug effect (within effect: F(1,19)=8.082, p=0.010, p_{perm}=0.001): While heart rate increased slightly under psilocybin, saline led to a decrease of heart rate. Comparison of respiration rate differences (Δpost-pre-injection, right panel) between psilocybin and saline treatment also demonstrated a significant drug effect (within effect: F(1,19)=6.611, p=0.019, p_{perm}=0.019): Respiration rate decreased

significantly stronger under psilocybin treatment then under saline treatment. **(D)** Both, mean heart (left panel) and respiration rate (right panel), calculated as the mean value of the baseline in session 1 and session 2, were significantly lower in ADE animals compared to control animals (heart rate: p_{perm}=0.0187; respiration rate: p_{perm}=0.0014).
ADE, alcohol deprivation effect rats (n=15); Con, control rats (n=6); Psi, psilocybin; Sal, saline; * p<0.05; ** p<0.01.

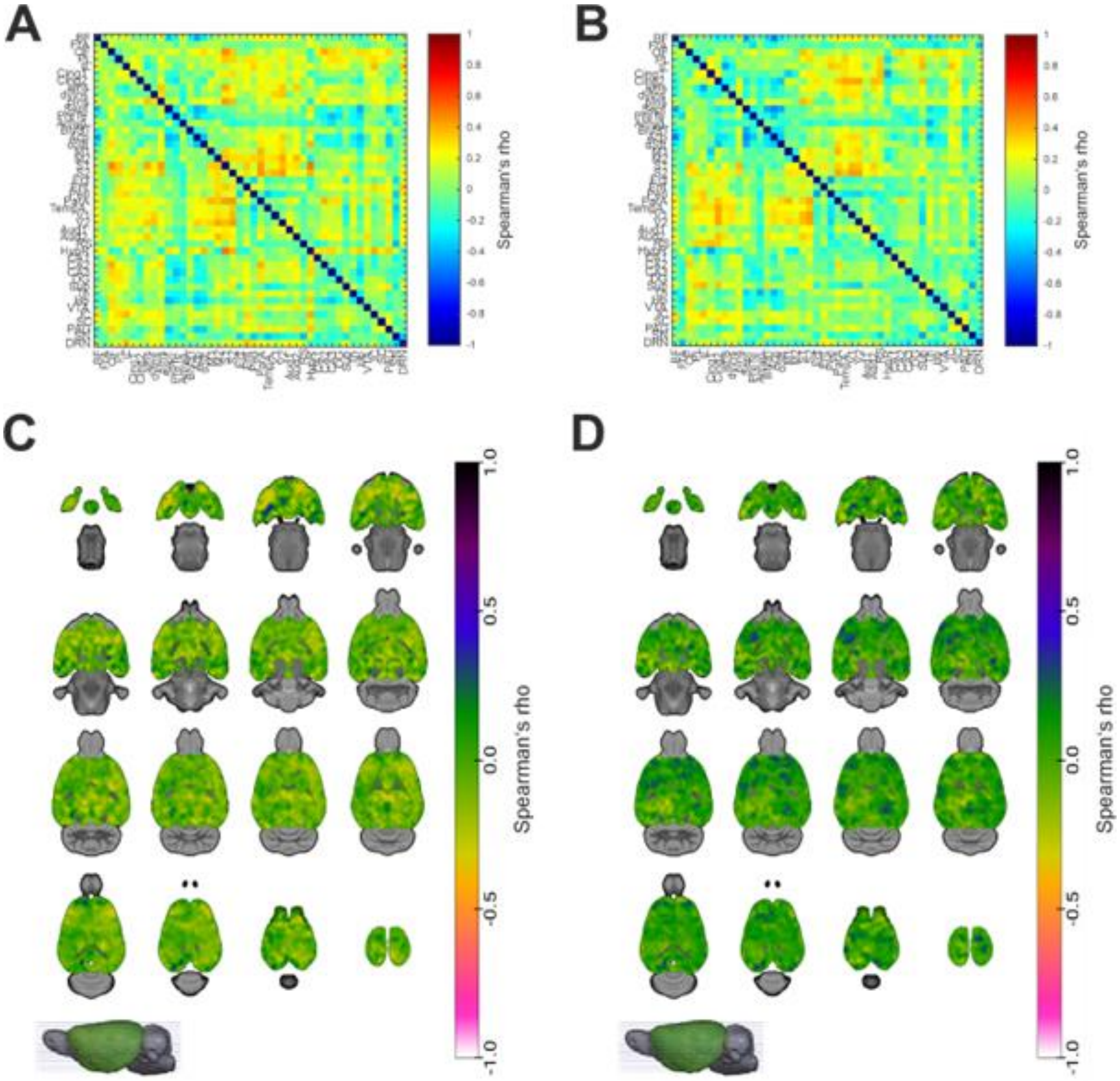

Fig. S8: Correlation of mean physiological parameters (mean respiration and mean heart rate) with region-of-interested (ROI)-based functional connectivity (FC) and global brain connectivity (GBC). Spearman's correlation coefficients between mean respiration rate **(A)** and mean heart rate **(B)** at baseline and ROI-based FC are illustrated in a matrix. Correlation coefficients were calculated with n=42 baseline sessions, associating the respective FC values between two ROIs with the mean respiration and heart rates at baseline. Of note, correlation values were relatively low for both comparisons and did not survive FDR correction (p_{FDR}>0.05, corrected for the number of connections n=946), arguing against a strong influence of physiological data on ROI-based FC. Spearman's correlation coefficients between physiological metrics at baseline and whole-brain voxel-wise GBC values are illustrated in **(C)** for mean respiration rate and **(D)** for mean heart rate. Correlation coefficients were calculated with n=42 baseline sessions, associating the respective GBC values with the baseline physiological metrics. Of note,

correlation coefficients were relatively low (greenish to light yellowish color) and no cluster survived multiple comparison correction even with a lenient cluster-defining threshold ($t>2.65$, corresponding to $p_{pt}<0.01$, $p_{FWE\ cluster-corrected}>0.05$), arguing against a strong influence of physiological data on GBC.
For region abbreviations, see Fig. 2.

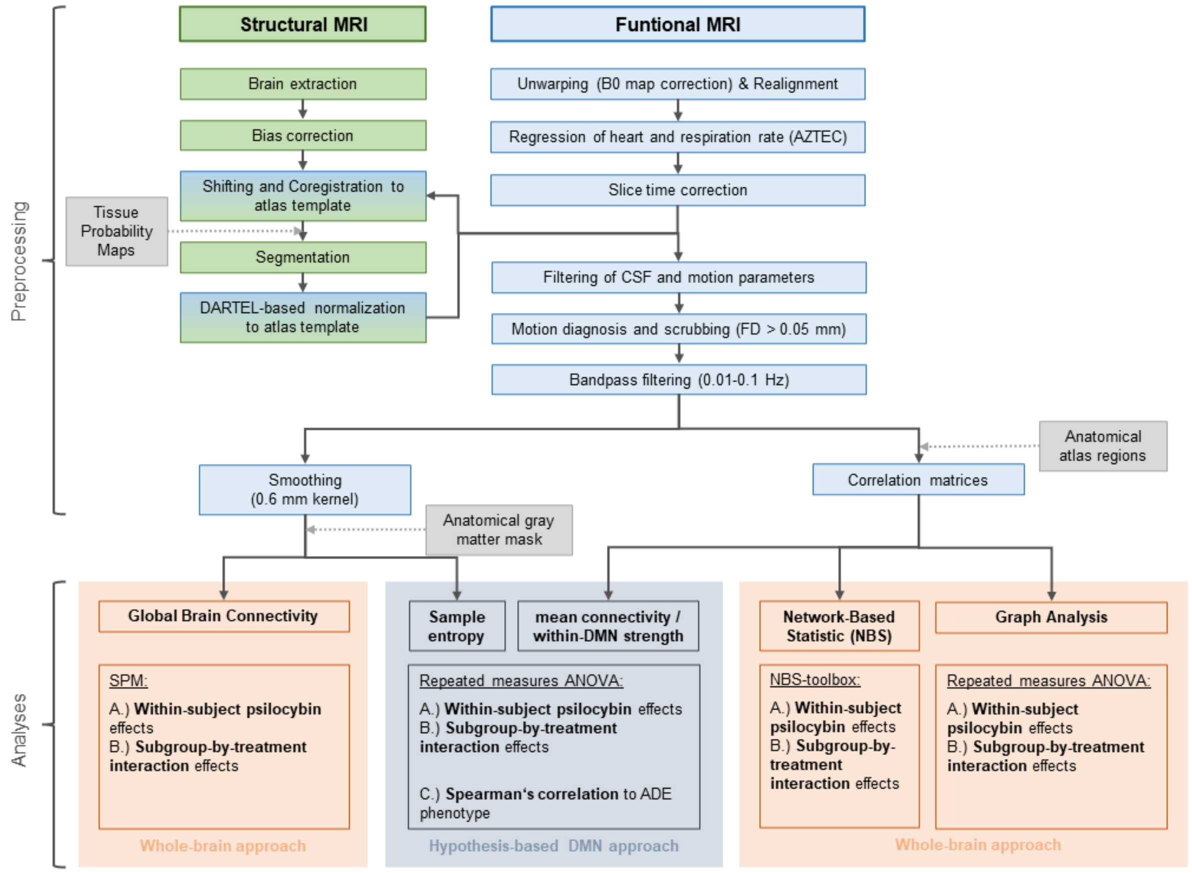

Fig. S9: Scheme of fMRI preprocessing steps and analytical approaches.
ADE, alcohol deprivation effect model; ANOVA, analysis of variance; Aztec, matlab tool for removal of cardiorespiratory noise (van Buuren et al., Hum Brain Mapp 2009); CSF, cerebrospinal fluid; DARTEL, Diffeomorphic Anatomical Registration using Exponentiated Lie algebra; DMN, default-mode network; FD, framewise displacement; Hz, hertz; SPM, standard parametric mapping software version SPM12, https://www.fil.ion.ucl.ac.uk/spm/software/spm12/
